# Culturally aware comparative evaluation of machine learning and deep learning models for cyberbullying detection

**DOI:** 10.3389/frai.2026.1871932

**Published:** 2026-10-20

**Authors:** Golam Md Mohiuddin, Md Shohel Sayeed, Ong Lee Yeng

**Affiliations:** 1 Faculty of Information Science and Technology, Multimedia University, Melaka, Malaysia; 2 Centre for Intelligent Cloud Computing, CoE for Advanced Cloud, Faculty of Information Science and Technology, Multimedia University, Melaka, Malaysia; 3 Centre for Advance Analytics, CoE for Artificial Intelligence, Faculty of Information Science and Technology, Multimedia University, Melaka, Malaysia

**Keywords:** culturally aware, cyberbullying detection, deep learning, machine learning, model evaluation

## Abstract

**Introduction:**

The rapid growth of social media has increased the prevalence of cyberbullying, raising serious psychological and social concerns. As abusive language varies across demographic and cultural groups, automated detection systems must be evaluated beyond overall accuracy to assess their effectiveness in identifying culturally sensitive forms of cyberbullying. This study proposes a culturally aware evaluation framework to compare traditional machine learning and deep learning models across four demographic-related categories: age, gender, religion, and ethnicity.

**Methods:**

Experiments were conducted using the IEEE DataPort Fine-Grained Balanced Cyberbullying Dataset. Traditional machine learning models were developed using Term Frequency–Inverse Document Frequency (TF-IDF) feature representations, while deep learning architectures included TextCNN, Bidirectional Long Short-Term Memory (BiLSTM), Bidirectional Gated Recurrent Unit (BiGRU), and CNN-BiLSTM. Model performance was assessed using accuracy, precision, recall, F1-score, confusion matrices, and class wise cultural sensitivity analysis.

**Results:**

The Extra Trees Classifier achieved the highest overall performance, with 97.79% accuracy, 97.82% precision, 97.79% recall, and 97.80% F1-score, followed by the Support Vector Machine, which achieved 93.30% accuracy and 93.28% F1-score. Among deep learning models, BiLSTM and BiGRU demonstrated competitive performance in capturing contextual patterns of abusive language. Class wise analysis showed that ethnicity and age were classified most accurately by the strongest traditional models, whereas gender remained the most challenging category across both machine learning and deep learning approaches.

**Discussion:**

The findings highlight the complementary strengths of traditional machine learning in identifying explicit lexical patterns and deep learning in capturing contextual representations of abusive language. Furthermore, variations in category specific performance demonstrate the importance of evaluating cyberbullying detection systems beyond aggregate metrics. This study provides insights into developing more robust, equitable, and culturally sensitive automated content moderation systems.

## 1 Introduction

In the digital era, social media platforms have become one of the most dominant forms of communication, enabling users to share information, express opinions, and interact globally in real time (Marengo et al., 2024). Despite these advantages, the rapid growth of online communication has also intensified harmful online behaviors, particularly cyberbullying. Cyberbullying refers to online harassment or abusive behavior conducted through digital platforms such as social media, messaging applications, and online forums (Ali and Nurjahan Begum Shahbuddin, 2022). It commonly affects adolescents and young adults and may involve offensive messages, hate speech, rumor spreading, impersonation, threats, and targeted humiliation (Bali et al., 2023). Recent global studies indicate that approximately 38–45% of adolescents worldwide have experienced some form of online harassment, highlighting cyberbullying as a growing public-health and societal concern (Faverio and Sidoti, 2024). Cyberbullying has been strongly associated with anxiety, depression, emotional distress, social withdrawal, reduced academic performance, and suicidal ideation, particularly among adolescents and young adults (Hani et al., 2019). The increased dependence on digital communication following the COVID-19 pandemic has further intensified exposure to harmful online interactions and psychological vulnerability among younger populations. Moreover, the effects of cyberbullying extend beyond victims alone, as bystanders may experience stress and moral conflict, while perpetrators themselves can exhibit long-term emotional instability and antisocial behavior (von Humboldt et al., 2025). These multidimensional psychological and social consequences reinforce the urgent need for reliable and scalable automated cyberbullying detection systems. Detecting cyberbullying automatically remains a challenging task due to the informal, noisy, and context-dependent nature of social-media text. Cyberbullying expressions frequently include sarcasm, implicit abuse, coded language, and culturally specific offensive terminology, making detection difficult using conventional text-classification methods (Chia et al., 2021). In addition, the way online abuse is expressed often differs across demographic and cultural groups, particularly in categories associated with age, gender, ethnicity, and religion. Consequently, cyberbullying detection systems that rely solely on overall classification accuracy may overlook important fairness and sensitivity issues across culturally diverse communities, potentially resulting in the under-detection of demographic-specific forms of online abuse.

Despite significant advances in automated cyberbullying detection, several important research gaps remain. First, many existing studies primarily emphasize improving overall classification accuracy while paying limited attention to how model performance varies across culturally and demographically sensitive cyberbullying categories. Second, traditional machine learning approaches frequently rely on sparse lexical feature representations, such as Term Frequency Inverse Document Frequency (TF-IDF), which do not explicitly model contextual semantics or sequential dependencies within text. Although these approaches have demonstrated competitive performance and strong interpretability, they are inherently limited in capturing implicit abusive language, sarcasm, and context-dependent expressions. More recently, deep learning architectures, including TextCNN, Bidirectional Long Short-Term Memory (BiLSTM), Bidirectional Gated Recurrent Unit (BiGRU), and hybrid CNN-BiLSTM networks, have demonstrated promising capabilities for learning contextual and sequential textual representations automatically, thereby improving the modeling of complex linguistic patterns in cyberbullying detection (Daraghmi et al., 2024). Nevertheless, relatively few studies have systematically compared traditional machine learning and deep learning approaches under a unified experimental framework using the same balanced fine-grained cyberbullying dataset. Furthermore, existing studies often rely on binary or coarse-grained classification tasks, providing limited understanding of demographic-specific cyberbullying behaviors involving age, gender, religion, and ethnicity. To address these limitations, this study presents a unified evaluation framework that systematically compares traditional machine learning and deep learning models using a balanced fine-grained cyberbullying dataset. Traditional machine learning models are trained using TF-IDF feature representations, whereas deep learning models learn contextual representations directly from textual sequences. This unified framework enables a systematic comparison of both learning paradigms while providing deeper insights into their effectiveness across culturally sensitive cyberbullying categories. Rather than proposing new culturally aware feature representations or bias-mitigation techniques, this study establishes a culturally aware evaluation framework for systematically assessing model performance across demographic-sensitive cyberbullying categories.

Figure 1 illustrates several common forms of cyberbullying, including harassment, electronic insults, happy beating, impersonation, cyber pursuit, and disclosure and hoax (López-Meneses et al., 2020). These forms represent different ways in which harmful or abusive behaviors can occur through digital communication, highlighting the importance of culturally aware evaluation when developing fair and effective automated cyberbullying detection systems.

**Figure 1:**
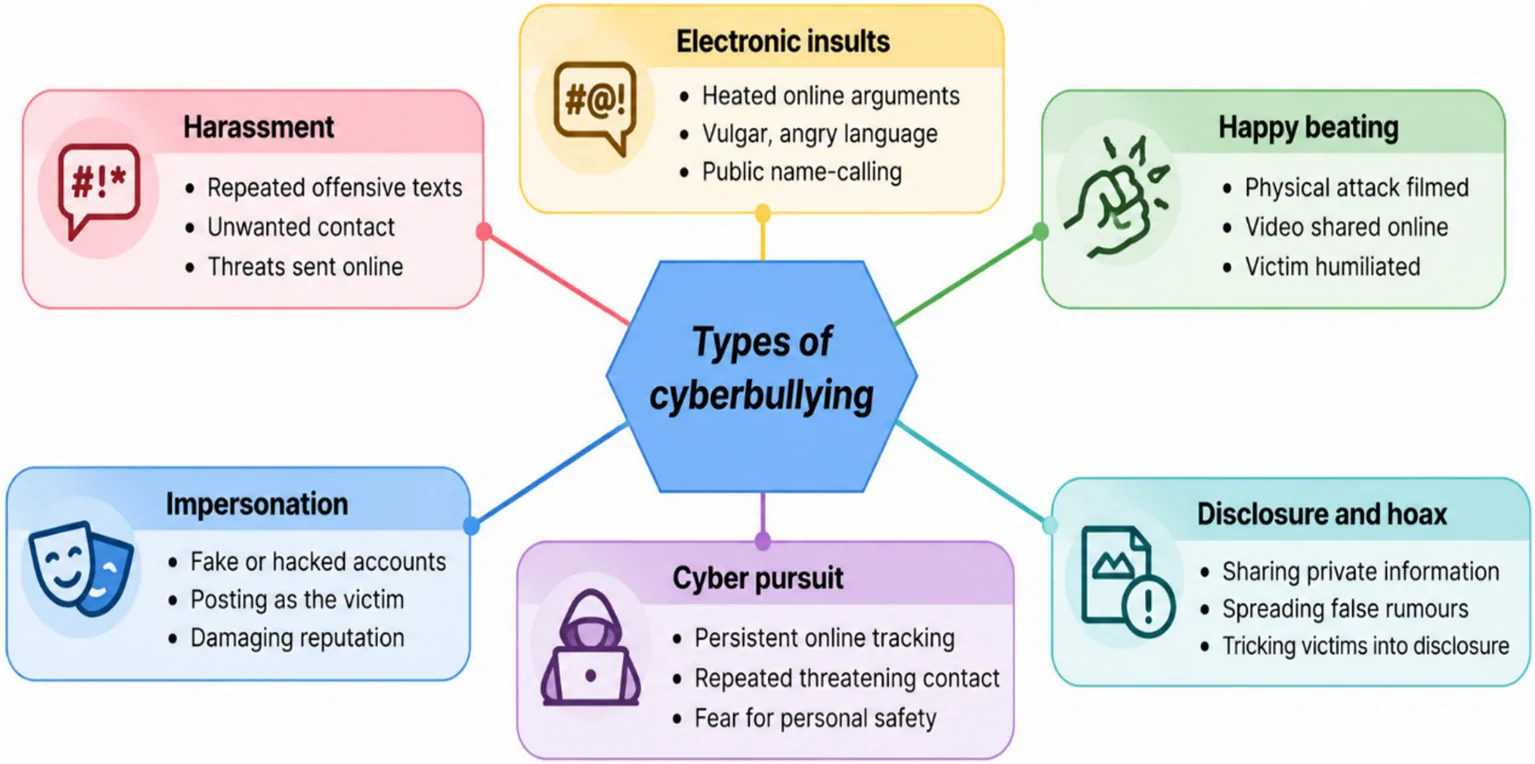
Types of cyberbullying (López-Meneses et al., 2020).

The primary objective of this research is to provide a comprehensive comparative evaluation of traditional machine learning and deep learning models for culturally aware cyberbullying detection. Unlike traditional studies that emphasize only global predictive accuracy, this work investigates how different learning paradigms perform across demographic-sensitive cyberbullying categories using a balanced fine-grained dataset. The main contributions of this study are summarized as follows:

- We propose a unified evaluation framework for culturally aware cyberbullying detection by systematically comparing traditional machine learning and deep learning models using a balanced fine-grained cyberbullying dataset.
- We evaluate nine traditional machine learning classifiers, including Support Vector Machine (SVM), Extra Trees, Random Forest, Logistic Regression, AdaBoost, XGBoost, K-Nearest Neighbors (KNN), Decision Tree, and Gradient Boosting, together with four deep learning architectures, namely TextCNN, BiLSTM, BiGRU, and CNN-BiLSTM, under a consistent experimental framework.
- We conduct a comprehensive quantitative evaluation using accuracy, precision, recall, and F1-score, together with category-wise performance analysis, confusion analysis, and feature importance analysis to better understand model behavior across demographic-sensitive cyberbullying categories.
- We provide comparative insights into the strengths and limitations of traditional feature-based learning and deep learning approaches, supporting the development of more effective and culturally aware cyberbullying detection systems.

Therefore, this paper is organized as follows. Section 2 reviews recent studies on cyberbullying detection, traditional machine learning, deep learning approaches, and the influence of cultural context on online abusive behavior. Section 3 presents the dataset, preprocessing procedures, feature representation methods, model architectures, hyperparameter optimization, and experimental setup. Section 4 reports the experimental results, comparative analyses, error analysis, and discussion. Finally, Section 5 concludes the paper and outlines future research directions for advancing culturally aware cyberbullying detection systems.

## 2 Literature review

Social networking platforms have become essential channels for communication, interaction, and information sharing. However, their widespread adoption has also facilitated harmful online behaviors, including cyberbullying, harassment, and abusive communication. These behaviors are particularly concerning among adolescents and young adults, who are highly active on social media platforms. Consequently, increasing research attention has been directed toward the automatic detection of cyberbullying using natural language processing, machine learning, and deep learning techniques. Recent studies have demonstrated the effectiveness of such computational approaches for identifying cyberbullying content and distinguishing among demographic-sensitive categories, including age, gender, religion, and ethnicity (Aliyeva and Yağanoğlu, 2024). The study contributors underscore the pivotal role of data collection and feature engineering in crafting robust machine learning, and deep learning techniques for cyberbullying prediction in social media environments. It advocates for the meticulous selection of discriminative features, incorporating sentiment analysis, user-specific attributes, and content-related markers, to enhance model efficacy. Feature selection algorithms, including chi-square, PCA, information gain, and Pearson correlation, are highlighted. The classification framework utilizes algorithms such as Support Vector Machine, Random Forest, K-Nearest Neighbor, Decision Tree, and Naive Bayes (Desai et al., 2021). The paper presents a detailed set of evaluation metrics, including accuracy, precision, recall, and f-measure, aimed at assessing model performance. The study concludes by articulating key research challenges and emphasizing the imperative for continued efforts in constructing highly effective cyberbullying detection models within the realm of online social networking sites (Kumar and Bhat, 2022). Although conventional machine learning have demonstrated competitive performance in cyberbullying detection, their effectiveness largely depends on feature engineering and the quality of textual representations. Widely adopted feature extraction techniques such as Bag-of-Words (BoW), N-Gram, and TF-IDF provide computationally efficient and interpretable representations but often fail to preserve contextual semantics and long-range linguistic dependencies. Consequently, these methods may struggle to accurately detect implicit abuse, sarcasm, figurative language, and culturally nuanced expressions that frequently occur in social media communication. These limitations have motivated increasing research interest in deep learning approaches capable of learning contextual representations directly from textual sequences.

One of the research highlights the harmful effects of cyberbullying on mental health, especially in young people, and tackles the widespread problem of cyberbullying on the internet and social media. Critiquing classical machine learning for their oversight of crucial features, the paper introduces a novel semi-supervised approach based on five categories: Senti-mental, Sarcastic, Syntactic, Semantic, and Social Features. Leveraging the bidirectional deep learning model BERT, the study attains promising accuracy, notably 91.90% with sentimental features alone. The results demonstrate BERT’s superiority over traditional models, with Support Vector Machine achieving 71.25% accuracy, surpassing Naive Bayes Classifier at 52.70%. The paper concludes by envisioning a comprehensive cyberbullying detection system, combining various models with BERT for real-world applications in identifying and reporting harmful content (Agrawal et al., 2024). Recent transformer-based architectures such as BERT, RoBERTa, and DistilBERT have demonstrated significant improvements in cyberbullying and hate-speech detection by capturing contextual semantics and implicit linguistic patterns more effectively than traditional machine learning approaches (Islam and Uddin, 2020). Besides transformer-based models, several lightweight deep learning architectures have also demonstrated strong performance in text classification tasks while requiring considerably lower computational resources. Text Convolutional Neural Networks (TextCNN) effectively capture local semantic patterns using convolutional filters, whereas Bidirectional Long Short-Term Memory (BiLSTM) and Bidirectional Gated Recurrent Unit (BiGRU) networks learn contextual information by processing textual sequences in both forward and backward directions (Chinmayi et al., 2026).

More recently, hybrid architectures such as CNN-BiLSTM have combined convolutional feature extraction with bidirectional sequence modeling to improve the representation of complex linguistic patterns in cyberbullying and abusive language detection. These architectures provide an attractive compromise between computational efficiency and contextual learning, making them suitable alternatives to transformer-based models for large-scale social media text classification (Hussain and Wahab, 2024). Unlike TF-IDF-based models that rely primarily on word frequency representations, transformer models utilize contextual embeddings that improve the detection of sarcasm, implicit abuse, and culturally nuanced expressions. Several recent studies reported that BERT-based frameworks achieved superior classification accuracy and robustness in social-media text analysis, particularly for detecting subtle and context-dependent cyberbullying behaviors. However, these deep learning approaches often require substantial computational resources and large annotated datasets, making traditional machine learning models still relevant for interpretable and computationally efficient cyberbullying detection systems. Using a system that includes preprocessing the dataset after importing the text dataset, the project focuses on cyberbullying detection using machine learning techniques. The authors incorporate emotional analysis and sentence size features derived from TF-IDF into the input information for machine learning. N-Gram techniques and the Blob library were utilized to analyze word combinations and identify misleading or noisy textual patterns, respectively. The proposed framework employed Support Vector Machine (SVM) and Neural Network classifiers for training and evaluation, with the combination of SVM and N-Gram features demonstrating the best performance. The study concluded that this framework improved cyberbullying detection and contributed to safer social media environments. However, the authors also noted that the limited size of the training dataset constrained performance and suggested that future studies should investigate deep learning approaches using larger datasets for enhanced detection accuracy (Ali and Syed, 2020). The study addresses the escalating issue of cyberbullying on social media platforms, particularly Facebook and Twitter, by employing machine learning algorithms for detection. The methodology encompasses Natural Language Processing and text preprocessing, involving the removal of irrelevant characters, tokenization, stemming, and more. Bag-of-Words (BoW) and TF-IDF techniques are then applied, and the study utilizes Decision Tree, Naive Bayes, Support Vector Machines, and Random Forest for categorization of remarks as either bullying or non-bullying. The conclusion emphasizes the growing significance of automated cyberbullying detection due to its prevalence on social media. The study explores the identification of cyberbullying posts using BoW and TF-IDF features with four machine learning algorithms, highlighting the effectiveness of Support Vector Machine (Bokolo and Liu, 2023).

Another study delves into the realm of cyberbullying detection through machine learning, utilizing labeled datasets obtained from the internet. Features like sentiment score, profanity, and sarcasm-related counts are extracted before applying preprocessing techniques, such as the removal of special characters, single characters, and stopwords, along with lowercase conversion. Employing models like Random Forest, Naïve Bayes, SVM, Logistic Regression, and Ensemble, the study evaluates their accuracy on four distinct datasets. The results reveal SVM and Ensemble as top-performing classifiers, achieving a noteworthy 79% average accuracy, followed closely by Logistic Regression at 78%, Random Forest at 76.7%, and Naïve Bayes at 76%. Notably, the study incorporates sarcasm as a critical aspect of cyberbullying detection, addressing a gap in previous research (Islam et al., 2024). Recent studies in cyberbullying detection have demonstrated the effectiveness of traditional supervised classifiers on demographic sensitive datasets. For instance, prior work analyzing 39,870 Twitter posts across distinct target categories (e.g., religion, age, gender, ethnicity) showed that ensemble models like Random Forest 94% and SVM 93% outperformed linear and instance-based algorithms such as Logistic Regression, Naïve Bayes, and KNN, underscoring the necessity of fine grained, category specific evaluation (Sayed et al., 2025). Furthermore, recent advancements in neural sequence learning and optimization techniques have introduced vital methodologies for refining text categorization. In the domain of network convergence, research has shown that standard gradient mechanics can be altered into sample-driven gradient descent models to accelerate text learning tasks (Ganie and Dadvandipour, 2023). To maximize context awareness across intricate text features, researchers have applied customized ensemble frameworks over pre-trained BERT platforms to systematically isolate localized harassment strings (Ganie and Dadvandipour, 2022). Related deep neural frameworks have also successfully expanded text categorization strategies by deploying multi-layered architectures designed to capture obsessive and highly repetitive semantic abusive behavior across diverse datasets (Ganie et al., 2021). These algorithmic optimizations illustrate the ongoing development of resilient deep sequence modeling frameworks.

In addition to improving predictive performance, recent research has increasingly emphasized fairness, robustness, and cultural awareness as essential characteristics of cyberbullying detection systems. Since abusive language varies across demographic and cultural groups, automated classifiers should not only achieve high overall accuracy but also maintain consistent performance across different categories of online harassment. Furthermore, the increasing deployment of artificial intelligence in social media moderation has highlighted the importance of developing secure and trustworthy models capable of resisting adversarial manipulation while minimizing demographic bias. Recent advancements in machine learning and cybersecurity also offer important insights that can strengthen the development of more robust and culturally aware cyberbullying detection systems. Islam et al. (2024) demonstrated that fairness-aware algorithms, such as Instant Runoff Voting (IRV), can mitigate biased decision-making an essential requirement for cyberbullying classification models operating across diverse demographic and cultural groups. Similarly, proposed an evolutionary defense framework based on bio-inspired reinforcement learning, illustrating how adaptive security mechanisms enhance model resilience against evasion and manipulation attempts (Kasri et al., 2025). Addressing concerns regarding system robustness, it has been shown that adversarial attacks targeting large language model based cyber threat intelligence systems can significantly compromise detection performance. Recent studies have increasingly explored context-aware deep learning approaches to improve cyberbullying detection on social media. One study employed a stacked BiGRU architecture integrated with attention and BERT to capture contextual and sequential characteristics of abusive online content, demonstrating the potential of combining recurrent networks with transformer-based representations for cyberbullying identification (Mali et al., 2025). Similarly, another study introduced an ELECTRA-based transformer incorporating part-of-speech information and enhanced tokenization to improve the recognition of contextual and subtle linguistic patterns in cyberbullying text (Azmi et al., 2025). These findings highlight the growing importance of context-sensitive deep learning architectures for addressing the limitations of conventional lexical feature-based approaches. Complementing these developments, the systematic review by Mohiuddin et al. (2025) emphasized the importance of cultural and religious context particularly within Muslim societies in shaping online abusive behavior, reinforcing the need for fairness, robustness, and contextual awareness in next-generation cyberbullying detection frameworks. Recent studies have also reported that annotation bias and demographic disparities may influence cyberbullying detection performance, emphasizing the need for fairness-aware evaluation across different social and cultural groups. Furthermore, multilingual cyberbullying datasets have highlighted the importance of evaluating detection models across diverse linguistic and cultural settings to improve robustness and generalizability. These observations further reinforce the need for culturally aware evaluation frameworks that assess model performance beyond overall classification accuracy. Despite considerable progress in automated cyberbullying detection, several research gaps remain. First, most previous studies evaluate either traditional machine learning or deep learning models independently, with limited comparative analysis under a unified experimental framework. Second, relatively few studies investigate classification performance across fine-grained demographic categories such as age, gender, religion, and ethnicity, despite their importance for culturally aware cyberbullying detection.

Finally, comprehensive evaluations that combine traditional machine learning, lightweight deep learning architectures, confusion analysis, and feature importance analysis remain scarce. Motivated by these research gaps, the present study systematically compares nine traditional machine learning classifiers and four deep learning architectures (TextCNN, BiLSTM, BiGRU, and CNN-BiLSTM) using a balanced fine-grained cyberbullying dataset. In addition to conventional performance metrics, the proposed framework incorporates culturally aware evaluation through category-wise analysis, confusion analysis, and feature importance assessment, thereby providing a more comprehensive understanding of model behavior across demographic-sensitive cyberbullying categories.

## 3 Methodology

This section describes the overall research methodology adopted in this study, including data acquisition, data preprocessing, feature representation, model development, and performance evaluation. Two modeling paradigms were investigated: traditional machine learning models based on TF-IDF feature representations and deep learning models that learn contextual representations directly from textual sequences. Figure 2 illustrates the overall workflow of the proposed research framework.

**Figure 2:**
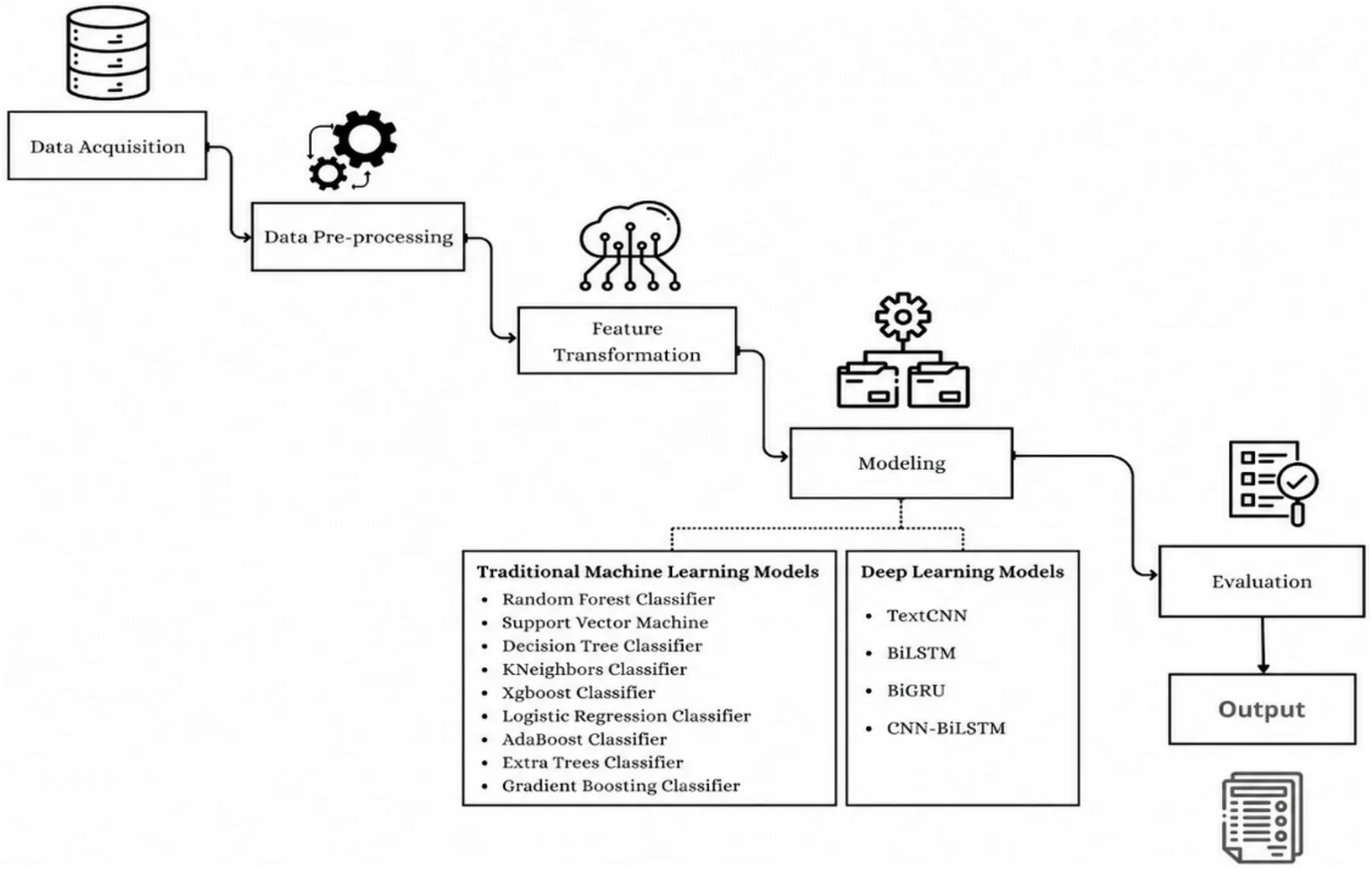
The flow diagram of the research.

### 3.1 Data acquisition

For this study, the Fine-Grained Balanced Cyberbullying Dataset was obtained from the IEEE DataPort repository. The dataset was originally introduced by Wang et al. (2020) in their 2020 study, SOSNet: A Graph Convolutional Network Approach to Fine-Grained Cyberbullying Detection, and was adopted as the primary data source for the machine learning experiments conducted in this research. The dataset provides a realistic and well-structured representation of fine-grained cyberbullying categories, making it suitable for evaluating machine learning models in culturally aware cyberbullying detection. Throughout the data acquisition process, ethical guidelines were observed by using an openly available dataset with appropriate attribution, thereby ensuring transparency, reproducibility, and scholarly integrity. The dataset consists of more than 47,000 English-language tweets, each annotated into one of six categories: age, gender, religion, ethnicity, other cyberbullying, and not cyberbullying. Each category contains approximately 8,000 instances, resulting in a balanced class distribution that minimizes class imbalance during model training and evaluation (Wang et al., 2021). Such balanced representation is particularly valuable for culturally aware analysis because it enables a fair comparison of machine learning performance across demographic-sensitive cyberbullying categories. Despite these strengths, several dataset limitations should be acknowledged. Although the dataset is balanced across demographic categories, annotation of offensive language inevitably involves subjective human judgment. Different annotators may interpret sarcasm, implicit aggression, coded language, or context-dependent expressions differently, potentially introducing inconsistencies in the assigned labels. Furthermore, certain forms of cyberbullying, particularly those targeting minority communities or relying on culturally specific expressions, may remain underrepresented. These factors may influence model generalization and contribute to variations in classification performance across different cyberbullying categories.

In addition, the dataset primarily consists of English-language Twitter (X) posts collected within a specific temporal and social context and therefore may not fully represent the linguistic and cultural diversity of global online communities. The original dataset creators did not publicly report the demographic characteristics of the annotators, such as their cultural, linguistic, or religious backgrounds. Consequently, the potential influence of annotator perspectives on labeling culturally sensitive content cannot be directly assessed in this study. Moreover, the dataset does not explicitly include multilingual or code-switched content; therefore, the proposed framework was evaluated only on English-language cyberbullying. Within this dataset, the “religion” category refers to abusive or discriminatory expressions targeting individuals or groups based on their religious identity or beliefs (e.g., Muslims, Christians, Jews, or Hindus), whereas the “ethnicity” category refers to abusive language directed toward individuals because of their ethnic origin, race, nationality, or cultural background, including racial slurs and xenophobic remarks. Consequently, the findings should be interpreted as an evaluation of culturally sensitive cyberbullying within English-language Twitter discourse, and caution should be exercised when generalizing the results to multilingual platforms or culturally distinct online communities. These limitations further highlight the importance of developing more culturally diverse datasets and conducting fairness-aware evaluations in future research.

#### 3.1.1 Dataset description from a culturally aware perspective

Table 1 shows that each cyberbullying category contains approximately 8,000 instances, resulting in an equal 16.7% distribution across all six classes. This balanced design is particularly important for culturally aware analysis, as it ensures that demographic-based bullying categories age, gender, religion, and ethnicity are represented equally. Such balance minimizes dataset-induced bias and allows the study to quantify cultural factors explicitly by comparing model behavior across different demographic groups. Prior to analysis, the dataset was examined for quality issues, and duplicates, contractions, emojis, URLs, newline characters, and stop words were removed. This ensured that the culturally associated linguistic patterns remained intact while the noise was reduced, enabling a more reliable evaluation of model performance.

**Table 1:** Culturally aware distribution of cyberbullying categories.

Cyberbullying category	Cultural/demographic factor
Age	Age-based harassment (e.g., “too old,” “kids”)
Gender	Gendered insults, sexism
Religion	Abusive or discriminatory expressions targeting religious identity or beliefs (e.g., Muslims, Christians, Jews, Hindus)
Ethnicity	Abusive expressions targeting ethnic origin, race, nationality, or cultural background, including racial slurs and xenophobic remarks
Other cyberbullying	General bullying not tied to a demographic
Non-cyberbullying	Neutral tweets, baseline class

Figure 3 presents the frequency distribution of culturally related keywords identified within the cyberbullying dataset. The keyword “muslim” shows the highest occurrence frequency, followed by other frequently observed terms such as “idiot” and “christian.” Additional keywords, including “terrorist,” “right,” “islamic,” “woman,” “islam,” “terrorism,” and “radical,” appear with comparatively lower frequencies. These frequencies represent the occurrence of specific terms within the collected social media content and should not be interpreted as direct evidence of cyberbullying exposure or targeting of any particular cultural or religious group. Instead, this keyword analysis provides insight into the raw lexical occurrence patterns and culturally associated expressions across the entire six-class dataset prior to model training.

**Figure 3:**
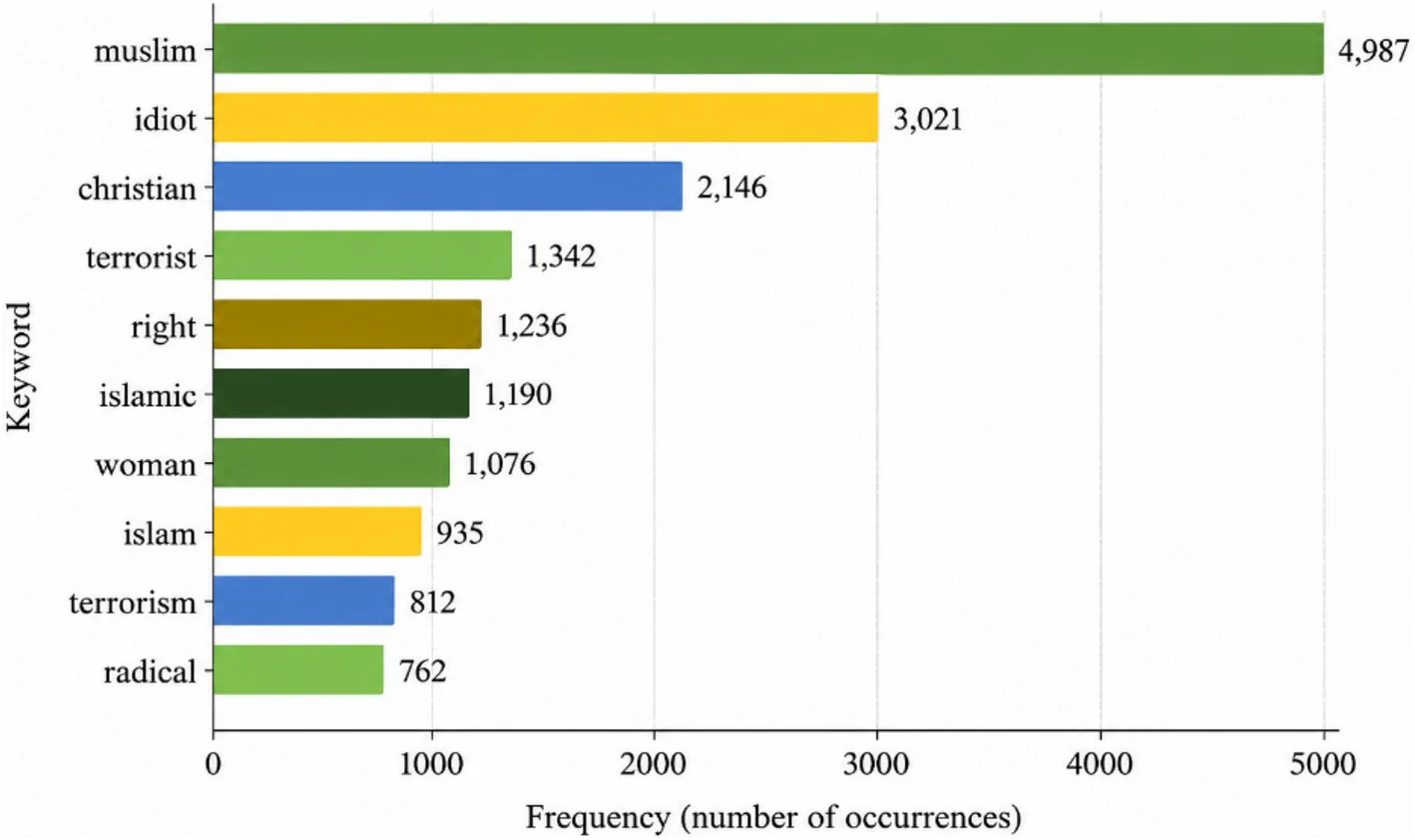
Frequency distribution of culturally related keywords in the cyberbullying dataset.

#### 3.1.2 Data preparation

Each instance in the dataset corresponds to a single tweet and consists of two primary attributes: the tweet text and its corresponding cyberbullying category illustrated in Table 2.

**Table 2:** Dataset attributes and sample entries.

Tweet	Level
Go back to your country!	Ethnicity
Only men should be engineers.	Gender
Stop pushing your religion on everyone.	Religion
You’re too old to use TikTok	Age
Nice photo!	Non-cyberbullying
You are so stupid, you’ll never amount to anything	Other cyberbullying

To further understand the nature and frequency of terms within the dataset, a word cloud was generated from the tweet content in Figure 4. This visualization highlights the most frequently occurring words within the dataset and provides an overview of common lexical patterns associated with different forms of cyberbullying. The keyword frequency analysis was conducted using raw occurrence counts of terms within the dataset. Therefore, the values presented in Figure 3 represent the number of appearances of each term and should not be interpreted as percentage distributions.

**Figure 4:**
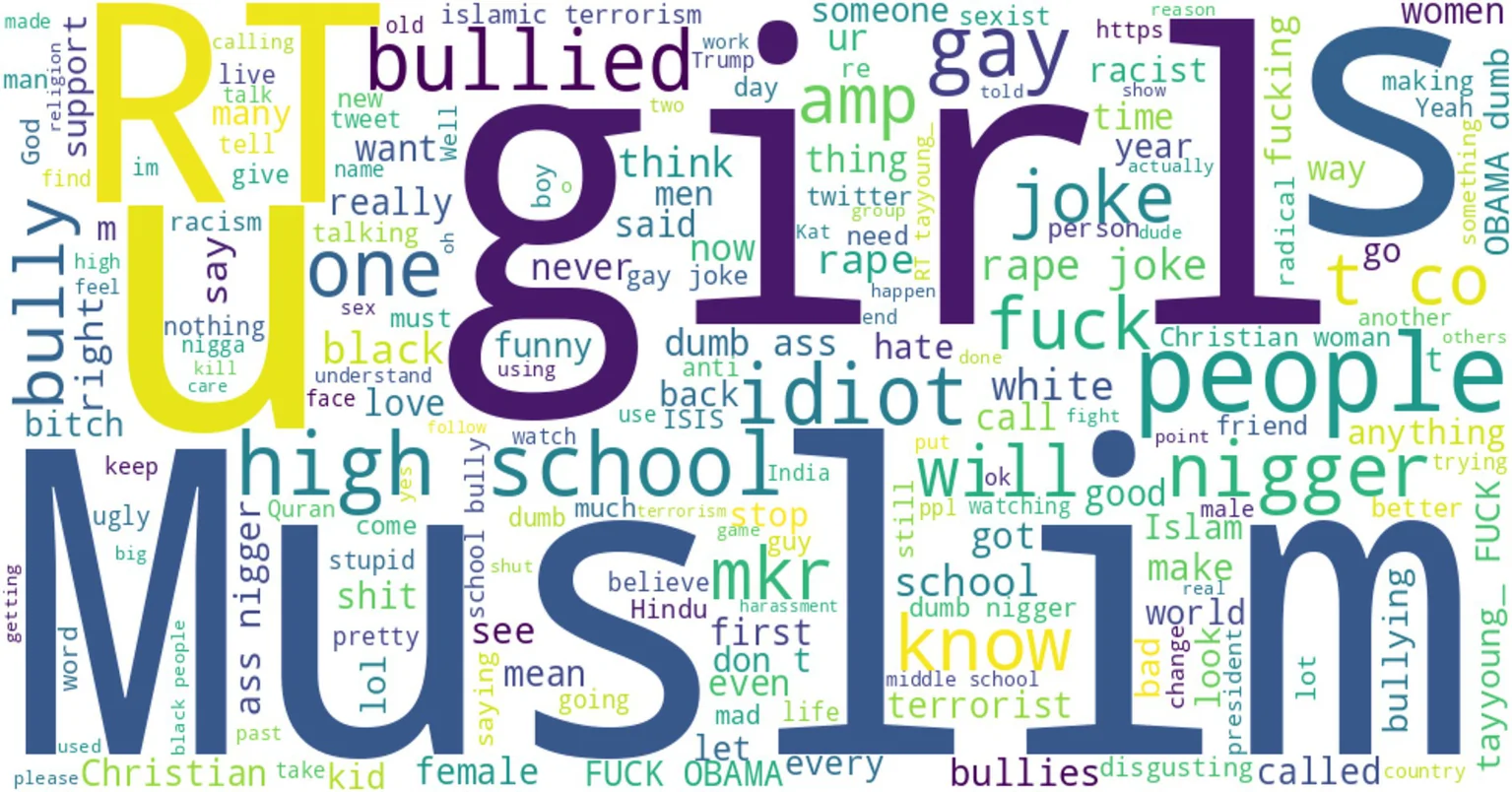
Frequently used words in cyberbullying tweets.

Data preparation is the process of converting raw data through pre-processing before being used in fitting and evaluating machine learning predictive systems (Rai et al., 2024). The data preparation process consisted of two major stages. The first stage focused on general data preprocessing, including duplicate removal, missing-value inspection, and text normalization. The second stage involved natural language processing techniques, including tokenization, lemmatization, stop-word removal, punctuation removal, and text cleaning. These preprocessing steps improved data quality and ensured that the textual representations were suitable for subsequent feature extraction and model training.

### 3.2 Data pre-processing

Data preprocessing stands as a pivotal stage in the machine learning workflow, wielding a profound impact on the generalization performance of supervised algorithms. The quality of datasets utilized in machine learning endeavors is integral to the production of reliable models that yield accurate results (Li et al., 2022). Through tasks such as handling missing values, normalizing features, encoding categorical variables, and ad-dressing imbalances, data preprocessing ensures that the input data is cleansed, standardized, and appropriately formatted for optimal model training (Borrohou et al., 2023). There are some common steps of data preprocessing and they are;

#### 3.2.1 Data cleaning

An important part of the preparation pipeline is cleaning the data, which focuses on rectifying errors and inconsistencies within a dataset to enhance its quality and re-liability. Tasks within data cleaning include handling missing values, removing duplicate records, addressing outliers, and correcting inaccuracies to ensure the dataset’s integrity (Swisstac Bravin, 2022).

#### 3.2.2 Data transformation

To make the dataset more suitable for machine learning analysis, the data trans-formation phase involved modifying the structure and format of the input data. Common practices included encoding categorical variables, normalizing features, and applying, mathematical operations to improve feature distribution (Rangineni et al., 2023). In addition to these standard transformations, special attention was given to handling missing values. The dataset was checked for any null or empty entries in both the tweet and label columns. Although the dataset was generally well-structured, a few records were found to contain missing or malformed values. These were removed using Pandas’ dropna() function to ensure data integrity and prevent errors during TF-IDF vectorization. For example, tweets without text or with undefined labels were excluded, as imputing or artificially filling such fields could introduce semantic distortion in a text-based classification task. This approach ensured that only clean, meaningful data was passed to subsequent stages of the machine learning pipeline.

#### 3.2.3 Data integration

The process of integrating data from many formats or sources to produce a single dataset is known as data integration. This consolidation facilitates a more comprehensive analysis by providing a holistic view of the data. Integration involves resolving schema mismatches, merging datasets with common identifiers, and handling inconsistencies across various sources (Volkan and Oktay, 2022).

#### 3.2.4 Data reduction

Data reduction optimizes dataset efficiency by minimizing volume while pre-serving critical information. Feature selection retains key variables, reducing dimensionality, and sampling methods generate representative subsets for resource-efficient analyses and robust machine learning model development (Hickman et al., 2020).

#### 3.2.5 Text pre-processing

In this study, given the text-based nature of the dataset, meticulous text preprocessing becomes imperative after the initial data preprocessing stages. This inclusive approach ensures the thorough structuring, cleaning, and standardization of textual data, establishing a foundation for robust analyses and precise model development within Natural Language Processing and text mining tasks. Specifically, structuring involved transforming all tweets into a consistent format by converting text to lower-case, ensuring proper spacing, and removing unnecessary punctuation. Cleaning procedures were applied to eliminate noisy elements such as URLs, hashtags, mentions for example @username, emojis, numbers, and other non-alphabetic characters that do not contribute to semantic meaning in cyberbullying classification. Additionally, standardization included expanding contractions for example “do not” → “do not,” “I’m” → “I am” and normalizing elongated words instant “soooo” → “so,” which helped unify di-verse expressions conveying the same intent. The task-specific nature of text preprocessing demands thoughtful consideration of techniques aligned with the analysis goals. Its effectiveness not only yields cleaner and standardized text data but also amplifies the performance of machine learning models and other text-centric analytical methods. This process is pivotal, contributing to heightened accuracy and efficiency in downstream tasks like sentiment analysis, text classification, and information retrieval within the realms of Natural Language Processing and text mining. Some of the text preprocessing steps include:

- Tokenization: Tokenization is a fundamental text-preprocessing step in natural language processing that decomposes textual content into smaller units, such as words or terms, which can subsequently be processed by machine learning and deep learning models. In cyberbullying detection, tokenization provides a structured representation of social media text and facilitates subsequent preprocessing and feature extraction. Recent cyberbullying detection studies have employed tokenization together with text cleaning, lowercase normalization, and stop word removal to prepare social media posts for classification (Al-Harigy et al., 2024).
- Stemming: Stemming is a text normalization technique that involves eliminating suffixes and, at times prefixes from words in order to truncate them to their base or root forms. The resulting stems may not always form valid words, but stemming is computationally efficient and facilitates the grouping of various word variations. For instance, stemming could derive “list” from “lists,” “listed,” and “listing.” However, it may also produce non-words, such as “lov,” from words like “loves,” “loving,” and “loved,” illustrating instances where the stemming process might yield less linguistically meaningful results (Pramana et al., 2022).
- Lemmatization: Lemmatization, unlike stemming, aims to reduce words to their base or dictionary forms, known as lemmas, without just removing suffixes. This process ensures that the resulting forms are valid words in the language. While lemmatization is more linguistically accurate, it often requires more computational resources compared to stemming. As an example, lemmatization could derive “go” from “going,” “went,” and “gone.” Unlike stemming, lemmatization typically produces valid words, contributing to linguistic precision in text-processing tasks. However, it may be computationally more intensive due to the need for access to a dictionary or linguistic resources (Hadiya, 2022).
- Removing Stopwords: Stopwords are common words that are often excluded from text processing as they carry little semantic meaning and can introduce noise to analyses. Removing stopwords is a crucial step in text preprocessing, enhancing the efficiency and accuracy of Natural Language Processing tasks. The list of stopwords typically includes words like “the,” “and,” “is,” etc. Although their removal may result in non-grammatical structures, it significantly streamlines analyses by eliminating frequently occurring yet non-informative terms. It would leave essential content words, yielding a more focused and meaningful representation of words (Faathima Fayaza and Farhath, 2021).

### 3.3 Feature transformation

This study employs the Term Frequency–Inverse Document Frequency (TF-IDF) technique to transform raw textual data into numerical feature vectors suitable for machine learning classification. TF-IDF is one of the most widely adopted feature extraction methods in natural language processing because it quantifies the importance of a term within a document while reducing the influence of commonly occurring words across the entire corpus. By combining Term Frequency (TF) and Inverse Document Frequency (IDF), TF-IDF generates discriminative feature representations that improve the ability of machine learning models to distinguish between different cyberbullying categories (Ogunleye and Dharmaraj, 2023). The TF-IDF weighting scheme is expressed in Equation 1:

TF−IDF(t,d)=TF(t,d)×IDF(t) (1)

where *TF* measures the frequency of a term within a document, and *IDF* quantifies the rarity of that term across the document collection.

The Term Frequency (TF) is calculated using Equation 2:

$$\mathrm{TF}(t,d)=\frac{\text{ft},d}{|d|}$$ (2)

where *ft,d* denotes the number of occurrences of term *t* in document *d*, and ∣*d*∣ represents the total number of terms contained in document *d*.

The Inverse Document Frequency (IDF) assigns lower weights to terms that occur frequently across the corpus while assigning higher weights to more discriminative terms. To moderate the influence of document frequency and avoid excessively large weights for common words, a logarithmic transformation is applied. The inverse document frequency (IDF) is calculated using Equation 3:

$$\mathrm{IDF}(t)=\log(\frac{N}{\mathrm{df}(t)})$$ (3)

where *N* represents the total number of documents in the corpus and *df*(*t*) denotes the number of documents containing term *t*. The logarithmic scaling suppresses the contribution of highly frequent words while emphasizing informative terms that better discriminate between cyberbullying categories.

In this study, the cleaned textual data were transformed into numerical features using a TF-IDF vectorization approach, following the feature-engineering pipeline illustrated in Figure 5. After splitting the dataset into training and testing partitions, each subset underwent a transformation process in which the text was normalized, tokenized, and stripped of noise such as URLs, mentions, emojis, punctuation, and stopwords. To prevent data leakage during experimentation, the TF-IDF vectorizer was fitted exclusively on the training dataset before transforming the testing dataset using the learned vocabulary and weighting scheme. During the fitting phase, the TF-IDF vectorizer learned the vocabulary and term-importance patterns from the training set, and this learned representation was subsequently applied to both bullying and non-bullying tweets. This process captured both individual keywords and short abusive phrases, generating a high-dimensional sparse matrix suitable for machine-learning classifiers. TF-IDF was selected because it has consistently demonstrated strong performance in text classification and cyberbullying detection while offering high computational efficiency and model interpretability. Compared with alternative feature extraction approaches such as Bag-of-Words (BoW) and Hash Vectorizer, TF-IDF provides more informative feature weighting by reducing the influence of ubiquitous terms and emphasizing discriminative lexical patterns. This characteristic is particularly beneficial for detecting culturally sensitive abusive expressions associated with demographic categories such as age, gender, religion, and ethnicity, thereby supporting the culturally aware evaluation framework proposed in this study. The TF-IDF vectorizer was configured using standard preprocessing settings appropriate for social media text classification. English stop words were removed to reduce linguistic noise, lowercase normalization was applied to ensure consistent textual representation, and both unigram and bigram features were extracted to capture individual keywords as well as short abusive phrases. Furthermore, sublinear term-frequency scaling was employed to reduce the influence of extremely frequent terms and improve the robustness of feature representations across diverse cyberbullying categories. To prevent data leakage, all text preprocessing operations, including tokenization, stop-word removal, lowercase normalization, and TF-IDF vectorization, were applied independently to the training and testing data within a Pipeline. The TF-IDF vectorizer, including the unigram and bigram vocabulary, was fitted only on the training set using fit_transform() and subsequently applied to the testing set using transform ().

**Figure 5:**
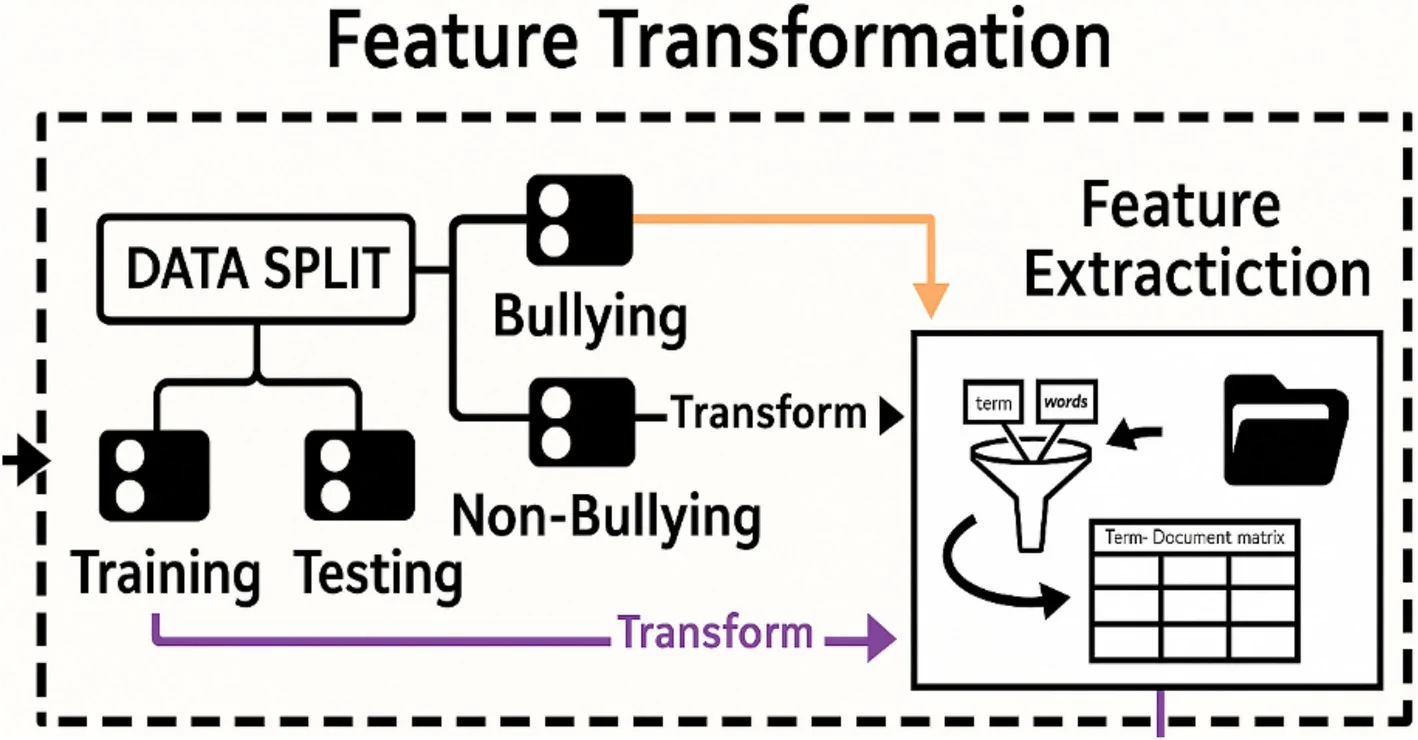
Feature transformation workflow using TF-IDF.

#### 3.3.1 Training and validation Split strategy

To evaluate the performance of various machine learning models in detecting cyberbullying, the dataset was randomly split into 80% for training and 20% for testing. This approach ensured that a significant portion of the data was used to train the models, while the remaining portion was reserved for evaluating their generalization ability on unseen data. The dataset was stratified according to the six cyberbullying categories (Age, Gender, Religion, Ethnicity, Other Cyberbullying, and Not Cyberbullying), thereby preserving the distribution of demographic-related categories across both the training and testing sets and supporting fair culturally aware evaluation. A fixed random seed value of 42 was used during dataset splitting and model initialization to ensure experimental reproducibility and consistency across repeated executions. This strategy allowed for a more accurate and fair assessment of each model’s performance, particularly in terms of their ability to detect nuanced patterns in text when transformed using TF-IDF. By maintaining consistent data distribution across both training and testing sets, we ensured that performance metrics such as accuracy, precision, and F1-score highlighted in Table 2 reflected the models’ true effectiveness in real world scenarios. All six categories defined in Table 1 (Age, Gender, Religion, Ethnicity, Other Cyberbullying, and Non-cyberbullying) were retained without merging, dropping, or exclusion throughout training, validation, and testing, and all reported evaluation metrics in Section 4 were computed from this same six-class prediction output.

### 3.4 Classification models

This study evaluates both traditional machine learning and deep learning models for fine-grained cyberbullying detection to provide a comprehensive comparison of different text classification approaches. The traditional machine learning models utilize TF-IDF feature representations, whereas the deep learning models learn semantic and contextual representations directly from word embeddings. The selected models were chosen because they represent widely adopted classification techniques with diverse learning characteristics, enabling a fair evaluation of their effectiveness in detecting culturally sensitive cyberbullying.

#### 3.4.1 Traditional machine learning models

In this research, a few machine-learning methods have been applied. The techniques utilized in this research are supervised classification machine learning algorithms since the dataset itself is a classification type of dataset.

##### 3.4.1.1 Random Forest (RF)

Random Forest (RF) is an ensemble-based supervised machine learning algorithm widely used for classification tasks due to its robustness, computational efficiency, and ability to reduce overfitting (Qaiser and Ali, 2018). The Random Forest classifier was optimized using GridSearchCV by exploring different combinations of ensemble size and splitting criteria. The final configuration selected by cross-validation used n_estimators = 100 and the Gini criterion. The model allowed unrestricted tree growth (max_depth = None) while maintaining the default minimum sample requirements for node splitting (min_samples_split = 2). In addition, the algorithm considered a subset of features during each split (max_features = ‘sqrt’) and utilized bootstrap sampling to improve ensemble diversity and generalization performance.

##### 3.4.1.2 Support Vector Machine (SVM)

Support Vector Machine (SVM) is a supervised machine learning algorithm widely used for classification tasks involving high-dimensional and sparse datasets such as TF-IDF text representations (Low et al., 2025). SVM operates by identifying an optimal hyperplane that maximizes the margin between different classes, making it highly effective for text classification problems (Al-Khowarizmi et al., 2023). In this study, the SVM model was configured using a radial basis function (RBF) kernel to capture nonlinear decision boundaries. The gamma parameter was set to ‘scale’ to control the influence range of support vectors, while the regularization parameter was configured as C = 1.0 to balance margin optimization and classification accuracy.

##### 3.4.1.3 Decision Tree (DT)

Decision Tree (DT) is a supervised machine learning algorithm widely used for classification and prediction tasks due to its interpretability and hierarchical decision-making structure (Wijayanti et al., 2022). The algorithm classifies data by recursively splitting features into decision nodes until a final class label is assigned (Alqowiy and Utami, 2025). In this study, the Decision Tree classifier was configured using the Gini impurity criterion with the ‘best’ splitter strategy to determine optimal feature splits. The model allowed unrestricted tree depth (max_depth = None) while maintaining the default minimum sample requirements for node splitting and leaf nodes (min_samples_split = 2 and min_samples_leaf = 1). In addition, the classifier considered all available features during split evaluation and applied balanced class weighting to reduce potential bias across cyberbullying categories.

##### 3.4.1.4 K Neighbors Classifier (KNN)

K-Nearest Neighbors (KNN) is a distance-based supervised machine learning algorithm commonly used for classification tasks due to its simplicity and effectiveness in pattern recognition (Yuvaraj et al., 2021). The algorithm predicts class labels based on the similarity between neighboring data points within the feature space. In this study, the KNN classifier was configured with n_neighbors = 5 using the Euclidean distance metric (*p* = 2) to measure similarity between TF-IDF feature vectors. Uniform weighting was applied so that each neighboring instance contributed equally during classification. In addition, the brute-force search algorithm was utilized for nearest-neighbor computation, while feature scaling and normalization were applied during preprocessing to improve distance-based classification performance in the high-dimensional text feature space (Zhang, 2022).

##### 3.4.1.5 AdaBoost classifier

AdaBoost is an ensemble-based boosting algorithm that improves classification performance by sequentially combining multiple weak learners and adjusting sample weights based on previous prediction errors (Islam and Uddin, 2020). In this study, a Decision Tree with a maximum depth of 1 was employed as the base estimator. The AdaBoost classifier was configured with n_estimators = 50 and a learning_rate = 1.0, while a fixed random state was used to ensure reproducibility. AdaBoost has also been investigated in previous cyberbullying detection studies as an ensemble learning approach for classifying abusive social-media content (Daniel et al., 2023).

##### 3.4.1.6 XgBoost classifier

XGBoost is a scalable ensemble-based machine learning algorithm built on gradient boosting decision trees and designed to improve predictive accuracy while reducing overfitting (Hornyák and Iantovics, 2023). The algorithm sequentially constructs decision trees by minimizing a predefined loss function and incorporates regularization techniques to enhance generalization performance. In this study, the XGBoost classifier was configured using the ‘gbtree’ booster with n_estimators = 100 and a learning_rate = 0.1 to balance learning stability and model complexity. The maximum tree depth was set to 6, while min_child_weight and gamma parameters were used to control overfitting during node splitting.

##### 3.4.1.7 Extra Trees Classifier (ETC)

Extra Trees Classifier (ETC) is an ensemble-based machine learning algorithm that constructs multiple randomized decision trees to improve classification accuracy and reduce variance (Bentéjac et al., 2020). Unlike traditional tree-based ensemble methods, ETC introduces additional randomness during node splitting by selecting cut points randomly and utilizing the entire training dataset rather than bootstrap sampling. This strategy improves computational efficiency and enhances model generalization performance (Peng et al., 2021). In this study, the ETC model was configured with n_estimators = 100 using the Gini impurity criterion for split evaluation. The classifier allowed unrestricted tree depth (max_depth = None) while maintaining default values for min_samples_split = 2 and min_samples_leaf = 1. In addition, the model used sqrt-based feature selection (max_features = ‘sqrt’), balanced class weighting, and a fixed random_state to ensure reproducibility. Parallel computation (n_jobs = −1) was also enabled to improve training efficiency when processing high-dimensional TF-IDF feature representations.

##### 3.4.1.8 Logistics Regression (LR)

Logistic Regression (LR) is a supervised machine learning algorithm commonly used for classification tasks due to its simplicity, interpretability, and effectiveness in handling linearly separable data (Ampomah et al., 2020). The algorithm estimates the probability of class membership using a logistic function and is widely applied in text classification and spam detection tasks (Livia Shreenithi et al., 2023). In this study, the Logistic Regression model was configured using L2 regularization with C = 1.0 to balance model complexity and generalization performance. The classifier included an intercept term (fit_intercept = True) and utilized the ‘lbfgs’ solver for optimization. In addition, the maximum number of iterations was set to 1,000 to ensure convergence during training, while the multiclass classification strategy was implemented using the one-vs-rest (ovr) approach with balanced class weighting to improve classification stability across demographic cyberbullying categories (Pattanaik et al., 2023).

##### 3.4.1.9 Gradient Boosting Classifier

Gradient Boosting Classifier is an ensemble-based machine learning approach that has been investigated for NLP-based cyberbullying detection alongside other conventional and ensemble classifiers, demonstrating its applicability to the classification of abusive social-media content (Akar, 2024). The algorithm iteratively minimizes a predefined loss function by correcting the errors of previously generated decision trees, making it highly effective for complex classification tasks (Dyer et al., 2022). In this study, the Gradient Boosting Classifier was configured using the deviance loss function with n_estimators = 100 and a learning_rate = 0.1 to balance model stability and generalization capability. The model utilized subsample-based training to reduce overfitting, while the Friedman mean squared error criterion was applied for split evaluation. In addition, the classifier-maintained default values for min_samples_split and min_samples_leaf, with a maximum tree depth of 3 and sqrt-based feature selection to improve robustness when handling high-dimensional TF-IDF feature representations. A fixed random_state was also applied to ensure reproducibility across experiments.

#### 3.4.2 Deep learning models

To complement the traditional machine learning models, this study evaluates four deep learning architectures: TextCNN, Bidirectional Long Short-Term Memory (BiLSTM), Bidirectional Gated Recurrent Unit (BiGRU), and CNN-BiLSTM. Unlike TF-IDF-based machine learning models, these architectures automatically learn semantic feature representations from textual sequences through trainable word embeddings. The selected models capture both local lexical features and long-range contextual dependencies, enabling a comprehensive comparison between traditional feature engineering and deep representation learning for culturally aware cyberbullying detection. All deep learning models were trained and evaluated using the same training and testing partitions to ensure a fair comparison with the traditional machine learning models.

##### 3.4.2.1 TextCNN

TextCNN is a convolutional neural network designed for text classification that automatically learns discriminative local features from word embeddings. In this study, tokenized tweets were converted into fixed-length sequences and processed through an embedding layer followed by one-dimensional convolution and max-pooling operations. The extracted features were passed to fully connected layers for classification. TextCNN was selected because of its computational efficiency and its ability to capture local contextual patterns and short abusive phrases commonly found in cyberbullying text.

##### 3.4.2.2 Bidirectional long short-term memory (BiLSTM)

BiLSTM extends the conventional LSTM by processing textual sequences in both forward and backward directions, allowing contextual information to be learned from preceding and succeeding words simultaneously. Tweets were represented using trainable word embeddings before being processed by a bidirectional LSTM layer and a fully connected classifier. BiLSTM was selected because it effectively captures long-range contextual dependencies and implicit semantic relationships that are difficult to represent using traditional TF-IDF features.

##### 3.4.2.3 Bidirectional gated recurrent unit (BiGRU)

BiGRU is a bidirectional recurrent neural network that employs a simplified gating mechanism to model sequential information efficiently. Compared with BiLSTM, BiGRU requires fewer parameters while maintaining strong contextual learning capability. In this study, embedded tweet sequences were processed using a bidirectional GRU layer followed by dense classification layers. The model was included to investigate whether a computationally efficient recurrent architecture could effectively identify contextual cyberbullying patterns.

##### 3.4.2.4 CNN-BiLSTM

CNN-BiLSTM combines convolutional neural networks with bidirectional LSTM networks to capture both local lexical features and long-range contextual information. The convolutional layer extracts informative local patterns from word embeddings, while the BiLSTM models sequential dependencies within the extracted features. The combined representations are then used for multi-class cyberbullying classification. This hybrid architecture was selected because it integrates the strengths of CNN and BiLSTM, enabling more effective detection of both explicit and context-dependent cyberbullying expressions.

### 3.5 Hyperparameter optimization and configuration

To ensure a fair and reproducible comparison across all classification models, this study adopted a combination of targeted hyperparameter optimization and standardized model configuration strategies. GridSearchCV optimization was applied to selected traditional machine learning classifiers, while other models were configured using predefined parameters based on established practices and empirical considerations. For the traditional machine learning models, targeted hyperparameter optimization was performed for Support Vector Machine (SVM), Random Forest (RF), and Extra Trees Classifier (ETC) using GridSearchCV with five-fold stratified cross-validation on the training dataset. These models were selected because their performance can be influenced by key hyperparameters such as regularization strength, tree complexity, and ensemble size. The optimal parameter combinations were selected based on the highest mean cross-validation accuracy. For the remaining machine learning classifiers, predefined configurations were adopted based on standard implementations, previous literature, and preliminary empirical evaluation. The deep learning models employed empirically selected hyperparameters based on widely adopted practices in text classification literature. The hyperparameter optimization strategies and final configurations are summarized in Tables 3, 4.

**Table 3:** Hyperparameter optimization and configurations of machine learning models.

Model	Parameter selection strategy	Hyperparameter search space/configuration	Final configuration
Support Vector Machine (SVM)	GridSearchCV	C: [0.1, 1.0, 10.0]; kernel: [‘linear’, ‘rbf’]; gamma: [‘scale’, ‘auto’]	kernel = ‘rbf’, C = 1.0, gamma = ‘scale’
Random Forest (RF)	GridSearchCV	n_estimators: [50, 100, 200]; criterion: [‘gini’, ‘entropy’]	n_estimators = 100, criterion = ‘gini’
Extra Trees Classifier (ETC)	GridSearchCV	n_estimators: [50, 100, 200]; max_depth: [None, 10, 20]; criterion: [‘gini’, ‘entropy’]	n_estimators = 100, criterion = ‘gini’, max_depth = None
Decision Tree (DT)	Predefined configuration	Standard parameter configuration	criterion = ‘gini’, splitter = ‘best’
K-Nearest Neighbors (KNN)	Predefined configuration	Standard parameter configuration	n_neighbors = 5, metric = ‘euclidean’, weights = ‘uniform’
AdaBoost Classifier	Predefined configuration	Standard parameter configuration	n_estimators = 50, learning_rate = 1.0
XGBoost Classifier	Predefined configuration	Empirically selected configuration	learning_rate = 0.1, max_depth = 6, n_estimators = 100
Gradient Boosting Classifier	Predefined configuration	Standard parameter configuration	n_estimators = 100, learning_rate = 0.1, max_depth = 3
Logistic Regression (LR)	Predefined configuration	Standard parameter configuration	solver = ‘lbfgs’, max_iter = 1,000, penalty = ‘l2’

**Table 4:** Hyperparameter configurations of deep learning models.

Model	Embedding dimension	Network configuration	Batch size	Epochs	Optimizer	Learning rate	Dropout
TextCNN	128	Conv1D (128 filters; kernel sizes = 3, 4, 5)	32	20	Adam	0.001	0.5
BiLSTM	128	64 units	32	20	Adam	0.001	0.5
BiGRU	128	64 units	32	20	Adam	0.001	0.5
CNN-BiLSTM	128	onv1D (128 filters; kernel size = 5) + Bidirectional LSTM (64 units)	32	20	Adam	0.001	0.5

The hyperparameter configurations were used consistently throughout all experiments to ensure reproducibility and a fair comparative evaluation of the investigated machine learning models.

All deep learning models were trained using the Adam optimizer with categorical cross-entropy as the loss function. Early stopping was employed to prevent overfitting by monitoring the validation loss, and the model with the best validation performance was retained for testing. The same training and testing partitions were used for all models to ensure a fair comparative evaluation. Therefore, the experimental framework does not represent exhaustive hyperparameter tuning for every classifier; instead, it combines targeted optimization for representative models with standardized configurations for comparative evaluation.

### 3.6 Performance measuring

The performance of both traditional machine learning and deep learning models was evaluated using accuracy, precision, recall, and F1-score. These evaluation metrics provide complementary perspectives on classification performance and enable a comprehensive comparison of the proposed models. Model evaluation plays a crucial role in assessing predictive effectiveness, supporting model selection, hyperparameter optimization, and understanding the trade-offs among different performance measures. Furthermore, it validates the ability of the developed models to generalize to unseen data, thereby ensuring reliable and robust cyberbullying detection. Evaluating models or algorithms after their application is essential to assess their effectiveness and guide decision-making in the machine learning process. It gives a quantitative indicator of performance, which helps with model selection, hyperparameter tweaking, and understanding the trade-offs between measures like as accuracy, precision, and F1 score. Evaluation also validates a model’s capacity to generalize to new data, facilitating iterative development and refinement throughout the machine learning workflow. These evaluation metrics were selected because they provide complementary perspectives on classification performance. Accuracy measures overall correctness, precision evaluates the reliability of positive predictions, recall assesses the ability to identify cyberbullying instances, and the F1-score balances precision and recall, making it particularly suitable for multiclass text classification.

- Accuracy: Accuracy is a key parameter in classification tasks, expressing the ratio of properly predicted cases to total instances in a dataset. It offers a basic overview of the model’s soundness, but might be deceptive in imbalanced datasets when one class dominates (Abdiyeva-Aliyeva et al., 2022).

Accuracy=(TP+TN)/(TP+FP+FN+TN)

- Precision: Precision is a metric that assesses the accuracy of positive predictions made by a classifier. It is the ratio of true positive predictions to the sum of true positives and false positives. Precision is especially valuable in scenarios where false positives are costly or impactful, emphasizing the precision of the model’s positive predictions (Naidu et al., 2023).

Precision=(TP)/(TP+FP)

- Recall: Recall, also known as sensitivity, measures the proportion of actual positive instances that are correctly identified by the classifier. It reflects the model’s ability to minimize false negatives and is particularly important in cyberbullying detection, where failing to identify abusive content may have serious consequences. The Recall score is calculated as

Recall=(TP)/(TP+FN)

- F1 Score: The F1 score provides a fair assessment of a classifier’s performance by taking the harmonic mean of accuracy and recall. It is especially helpful when there is an unequal distribution of classes and takes into account both false positives and false negatives (Foody, 2023). The F1 score is calculated as

$$\mathrm{F1}\;\text{Score}=2^{*}({\text{Recall}}^{*}\text{Precision})/(\text{Recall}+\text{Precision})$$

In addition to quantitative evaluation metrics, confusion matrices were analyzed to visualize class-wise prediction performance and identify common misclassification patterns among demographic categories such as age, gender, religion, and ethnicity. This analysis provides additional insights into the strengths and limitations of each classification model.

## 4 Results and discussion

This section presents the experimental results and comparative analysis of both the traditional machine learning and deep learning models developed for fine-grained cyberbullying detection. The performance of all models was evaluated using accuracy, precision, recall, and F1-score to provide a comprehensive assessment of their classification capability. First, the performance of the traditional machine learning models using TF-IDF feature representations is presented, followed by the evaluation of the deep learning models. Subsequently, a comparative analysis between the two learning paradigms is conducted, together with confusion matrix analysis, cultural and demographic sensitivity analysis, and a discussion of the strengths, limitations, and practical implications of the proposed approaches.

### 4.1 Performance comparison of traditional machine learning models

Table 5 summarizes the performance of the nine traditional machine learning classifiers evaluated using TF-IDF feature representations. The evaluation was conducted using accuracy, precision, recall, and F1-score to provide a comprehensive assessment of classification performance. As shown in Table 5, the Extra Trees Classifier achieved the highest overall performance, obtaining an accuracy of 97.79%, followed by Support Vector Machine (93.30%) and Random Forest (91.66%). In contrast, K-Nearest Neighbors (KNN) produced the lowest performance, with an accuracy of 63.71%, indicating that distance-based classifiers are less suitable for high-dimensional sparse TF-IDF feature spaces. In contrast, K-Nearest Neighbors (KNN) produced the lowest performance, with an accuracy of 63.71%, indicating that distance-based classifiers are less suitable for high-dimensional sparse TF-IDF feature spaces.

**Table 5:** Performance metrics of traditional machine learning models for cyberbullying detection using TF-IDF features.

Model	Accuracy (%)	Precision (%)	Recall (%)	F1-score (%)
Random Forest	91.66	91.94	91.34	91.64
SVM	93.30	93.35	93.30	93.28
Decision Tree	87.49	87.52	87.46	87.49
KNN	63.71	78.65	46.22	58.22
XGBoost	86.14	86.73	85.44	86.08
Logistic Regression	85.00	85.55	84.34	84.94
AdaBoost	80.97	82.24	79.37	80.78
Extra Trees	97.79	97.82	97.79	97.80
Gradient Boosting	81.46	83.70	78.75	81.15

The results indicate that tree-based ensemble methods consistently outperformed the remaining traditional classifiers, with Extra Trees and Random Forest achieving the highest overall performance. The superior performance of the Extra Trees Classifier can be attributed to its highly randomized tree construction strategy, which increases model diversity and reduces variance, thereby improving generalization when learning from sparse TF-IDF representations. Similarly, Random Forest benefits from ensemble averaging across multiple decision trees, producing robust predictions while reducing the risk of overfitting. Support Vector Machine also demonstrated competitive performance, achieving the second-highest accuracy. This result is consistent with previous studies because SVM is particularly effective in high-dimensional text classification problems. By maximizing the decision margin between classes, SVM efficiently separates cyberbullying categories represented by sparse TF-IDF vectors and maintains strong generalization performance even when many irrelevant features are present. In contrast, the boosting-based classifiers exhibited comparatively lower performance. Although XGBoost achieved competitive results, AdaBoost and Gradient Boosting showed reduced effectiveness on the cyberbullying dataset. TF-IDF produces high-dimensional sparse feature vectors in which many informative words occur infrequently, while social media posts often contain slang, misspellings, implicit abuse, and culturally dependent expressions. During boosting, AdaBoost progressively increases the weights of previously misclassified samples. These difficult samples frequently correspond to noisy or ambiguous tweets rather than clear decision boundaries, causing the classifier to overemphasize atypical observations and reducing its ability to generalize to unseen data. Ensemble methods based on independently constructed decision trees, such as Random Forest and Extra Trees, are less sensitive to this issue because their predictions are aggregated across multiple diverse learners.

The Decision Tree and Logistic Regression classifiers achieved moderate performance, whereas KNN recorded the lowest accuracy among all evaluated models. The poor performance of KNN is mainly attributable to the curse of dimensionality, where Euclidean distances become less discriminative as feature dimensionality increases. Consequently, neighborhood relationships in sparse TF-IDF space fail to accurately reflect semantic similarity between tweets, limiting the effectiveness of distance-based classification for cyberbullying detection. Overall, the comparative results presented in Figure 6 demonstrate that ensemble tree-based methods are the most effective traditional machine learning approaches for fine-grained cyberbullying detection using TF-IDF features, with the Extra Trees Classifier providing the best balance between predictive accuracy, robustness, and generalization across the evaluated cyberbullying categories.

**Figure 6:**
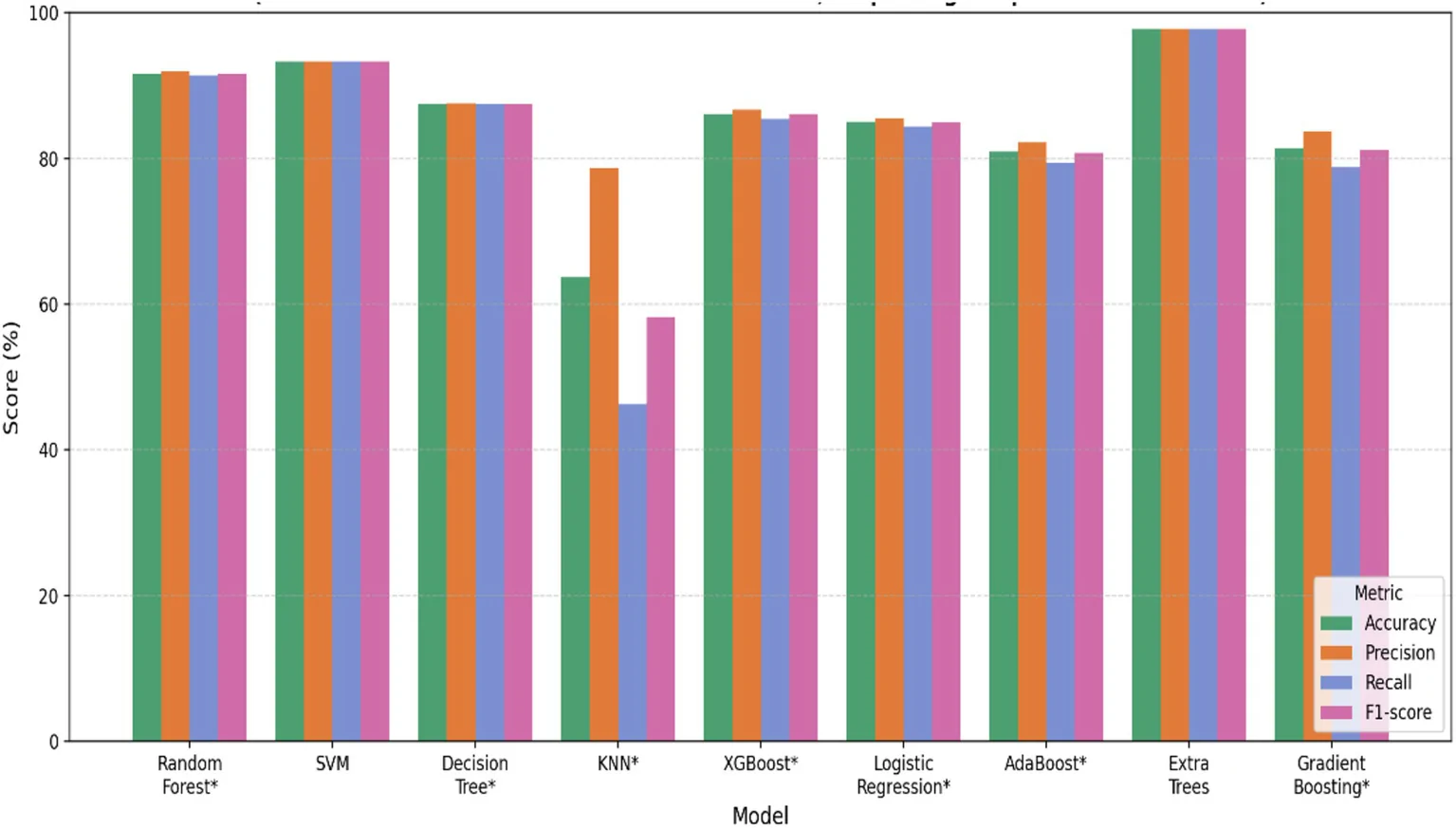
The models’ performance evaluation using TF-IDF.

### 4.2 Performance comparison of deep learning models

Table 6 summarizes the performance of the four deep learning models evaluated for fine-grained cyberbullying detection. The models were assessed using accuracy, precision, recall, and F1 score on the test dataset. Unlike the traditional machine learning models that relied on TF-IDF feature representations, the deep learning models learned dense semantic representations through trainable word embeddings, enabling them to capture contextual information and sequential dependencies within cyberbullying-related text.

**Table 6:** Performance metrics of deep learning models for cyberbullying detection.

Model	Accuracy (%)	Precision (%)	Recall (%)	F1-score (%)
BiLSTM	90.50	67.44	74.43	70.47
BiGRU	90.49	67.14	74.83	70.43
CNN-BiLSTM	81.09	65.88	79.36	68.24
TextCNN	78.75	67.30	81.27	68.92

As shown in Table 6 and illustrated in Figure 7, the BiLSTM model achieved the highest overall classification performance, obtaining an accuracy of 90.50% and the highest F1 score (70.47%). The strong performance of BiLSTM can be attributed to its bidirectional architecture, which captures contextual dependencies from both preceding and succeeding words. Such contextual modeling enables the network to better understand implicit abusive expressions and long-range semantic relationships that frequently occur in cyberbullying messages. The BiGRU model produced performance comparable to BiLSTM, achieving an accuracy of 90.49% and F1 score of 70.43%. Although GRU employs a simpler gating mechanism than LSTM, it effectively models sequential textual information while requiring fewer parameters, resulting in similar predictive performance with lower computational complexity. The slightly higher recall obtained by BiGRU indicates that it identified a larger proportion of cyberbullying instances across multiple categories. In contrast, TextCNN and CNN-BiLSTM achieved lower overall accuracy than the recurrent architectures. TextCNN obtained an accuracy of 78.75%, while CNN-BiLSTM achieved 81.09%. Nevertheless, both models demonstrated relatively high recall values, indicating their ability to identify a broad range of cyberbullying instances. However, this increase in recall was accompanied by lower precision, suggesting that these architectures produced more false-positive predictions. This behavior is expected because convolutional networks primarily learn local lexical patterns and short contextual windows, which may be insufficient for accurately modeling the complex semantic dependencies and implicit abusive expressions commonly observed in social media text. Overall, the experimental results indicate that recurrent neural network architectures outperformed convolution-based deep learning models for fine-grained cyberbullying detection. The superior performance of BiLSTM and BiGRU demonstrates the importance of modeling bidirectional contextual information when identifying subtle and context-dependent cyberbullying expressions. Although TextCNN and CNN-BiLSTM effectively captured local textual features, their comparatively lower precision and F1-score suggest that sequential contextual modeling remains more suitable for this classification task.

**Figure 7:**
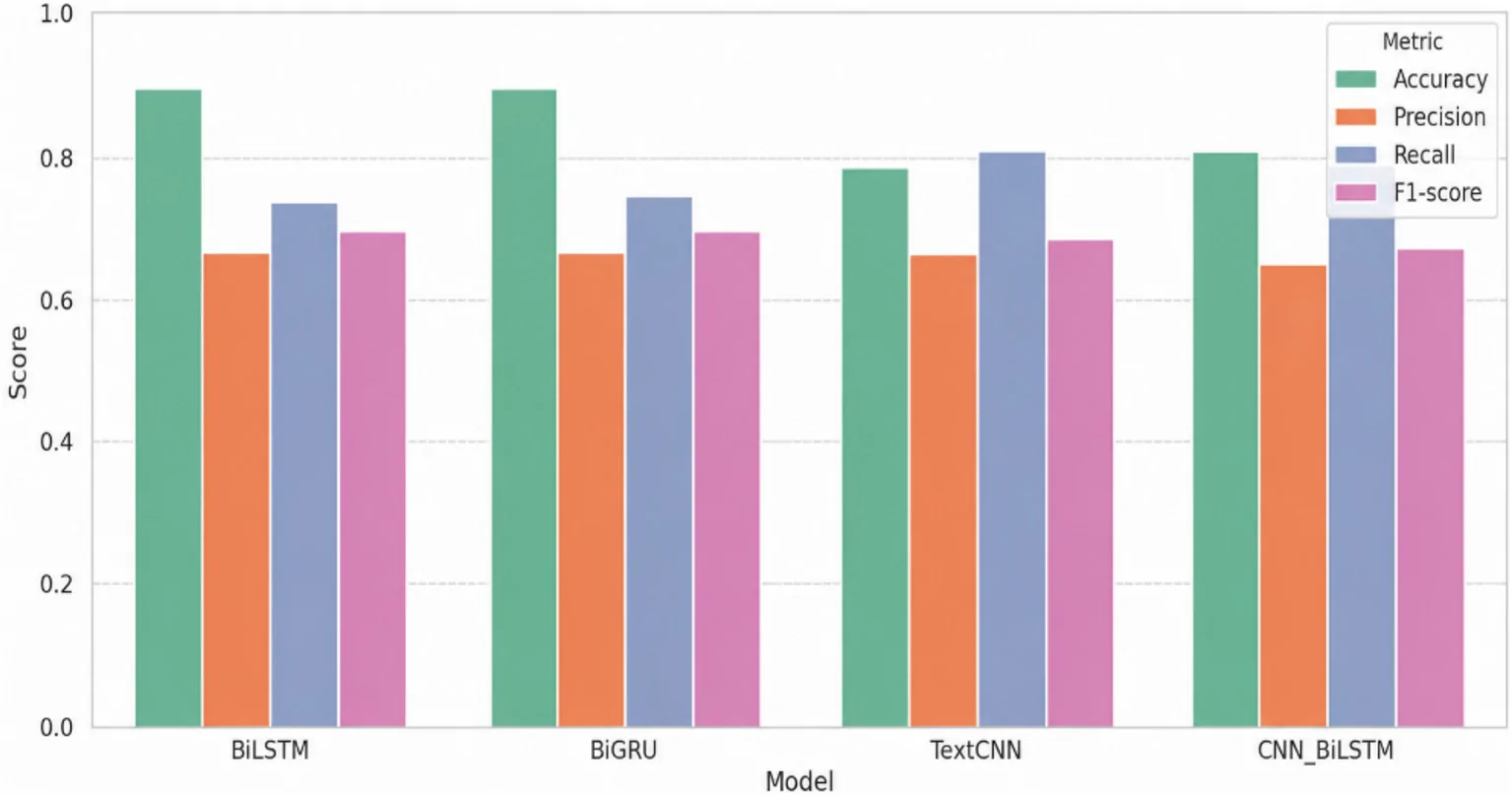
Performance comparison of deep learning models.

### 4.3 Comparative analysis between machine learning and deep learning

Table 7 compares the best-performing traditional machine learning models with the four deep learning models. The comparison highlights the strengths and limitations of each learning paradigm in terms of predictive performance, computational efficiency, model interpretability, and the ability to capture contextual information from cyberbullying-related text.

**Table 7:** Comparative performance of machine learning and deep learning models.

Model	Category	Accuracy (%)	Precision (%)	Recall (%)	F1-score (%)
Extra Trees	Machine learning	97.79	97.82	97.79	97.80
Support Vector Machine	Machine learning	93.30	93.35	93.30	93.28
Random Forest	Machine learning	91.66	91.94	91.34	91.64
BiLSTM	Deep learning	90.50	67.44	74.43	70.47
BiGRU	Deep learning	90.49	67.14	74.83	70.43
CNN-BiLSTM	Deep learning	81.09	65.88	79.36	68.24
TextCNN	Deep learning	78.75	67.30	81.27	68.92

The results reveal that the Extra Trees Classifier achieved the highest overall performance, obtaining an accuracy of 97.79% and an F1-score of 97.80%, outperforming all evaluated deep learning models and all other traditional machine learning models. Support Vector Machine also demonstrated strong performance (93.30% accuracy, 93.28% F1-score), indicating that traditional machine learning models remain highly effective when combined with discriminative TF-IDF feature representations. The strong performance of these models suggests that explicit lexical patterns provide sufficient discriminatory information for identifying many forms of cyberbullying within the balanced dataset. Among the deep learning models, BiLSTM achieved the highest overall accuracy (90.50%) and F1 score (70.47%), followed closely by BiGRU. The recurrent architectures consistently outperformed TextCNN and CNN-BiLSTM because bidirectional recurrent networks are capable of modeling sequential dependencies and contextual relationships between words. This enables them to better capture implicit abusive expressions, contextual semantics, and long-range linguistic dependencies that are difficult to represent using sparse TF-IDF vectors. From a computational perspective, traditional machine learning models were considerably more efficient than the deep learning architectures. Training the TF-IDF-based classifiers required substantially less computational time and memory while producing highly competitive classification performance. In contrast, the deep learning models required iterative optimization over multiple epochs and a larger number of trainable parameters, resulting in increased computational cost. Consequently, traditional machine learning models are more suitable for resource-constrained environments and applications requiring rapid model deployment.

However, the two learning paradigms exhibit different strengths. Traditional machine learning models provide greater interpretability because the contribution of individual TF-IDF features can be analyzed directly through feature importance scores or model coefficients. This transparency facilitates the identification of culturally associated lexical patterns associated with demographic categories such as age, gender, religion, and ethnicity. Conversely, deep learning models automatically learn distributed semantic representations that capture contextual meaning, sequential dependencies, and implicit abusive language without requiring manually engineered features. Such contextual learning is particularly beneficial for recognizing subtle cyberbullying expressions involving sarcasm, implicit harassment, or ambiguous linguistic constructions. The comparative analysis demonstrates that traditional machine learning models achieved the highest predictive performance on the current balanced dataset, whereas deep learning models offered superior contextual modeling capabilities. These findings suggest that the selection of an appropriate classification approach should depend on the specific application requirements. When computational efficiency, interpretability, and reproducibility are prioritized, TF-IDF-based ensemble learning remains an effective solution. Conversely, when detecting semantically complex and context-dependent cyberbullying expressions, recurrent deep learning architectures provide greater potential for capturing nuanced linguistic patterns. Future research may further improve detection performance by integrating interpretable statistical features with contextual neural representations within hybrid machine learning frameworks.

### 4.4 Confusion matrix analysis of the best models

Although overall evaluation metrics summarize classification performance, they do not reveal class-specific prediction behavior or systematic misclassifications. Therefore, confusion matrices were analyzed to examine prediction patterns across culturally sensitive cyberbullying categories. This analysis provides deeper insight into the strengths and limitations of both traditional machine learning and deep learning models. Based on the results presented in Sections 4.1–4.3, the Extra Trees Classifier and Support Vector Machine (SVM) were selected as the best-performing traditional machine learning models, whereas BiLSTM and BiGRU were selected as the best-performing deep learning models. Their corresponding confusion matrices are presented in Figures 8–11.

**Figure 8:**
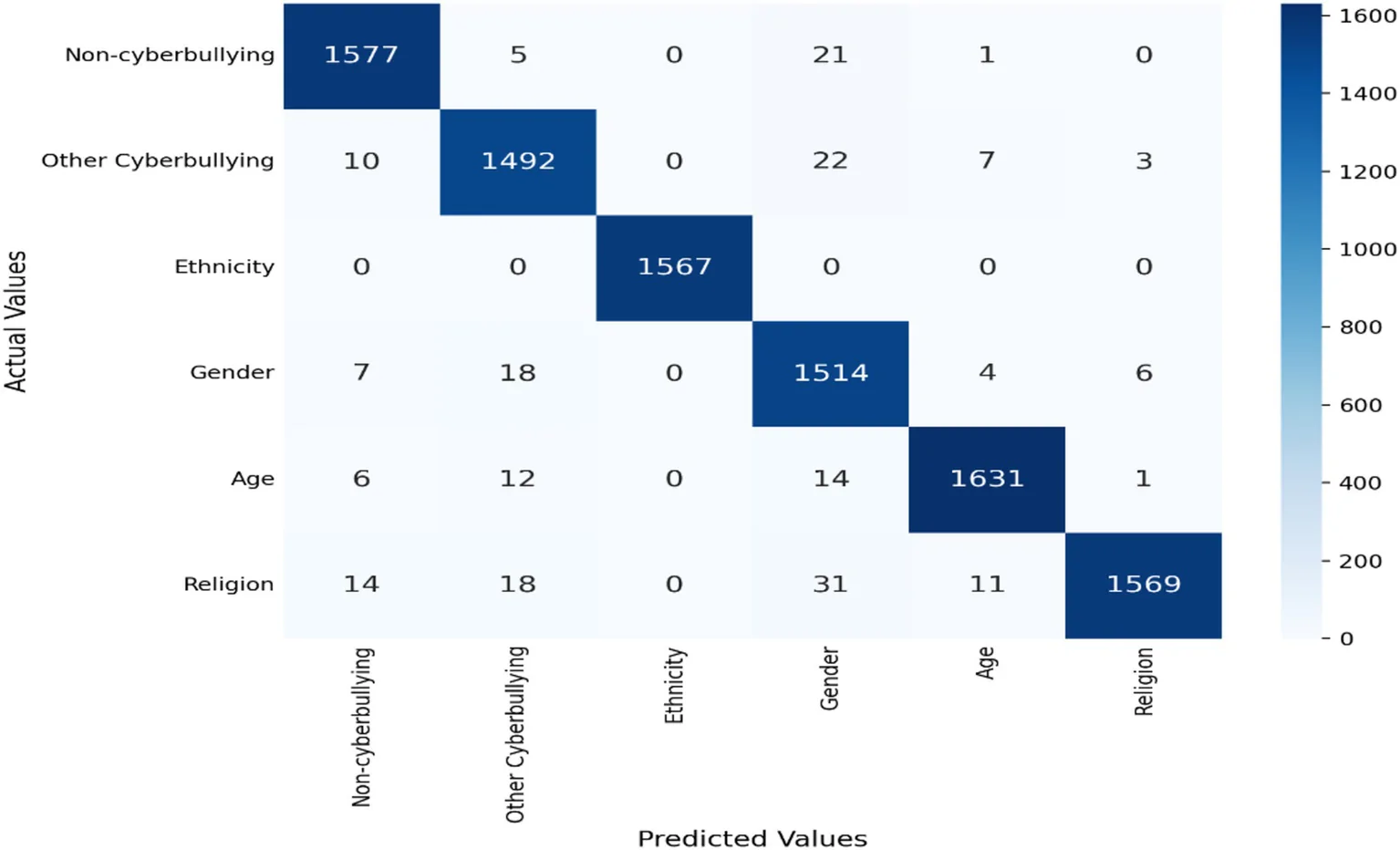
Six-class confusion matrix of the Extra Trees Classifier.

**Figure 9:**
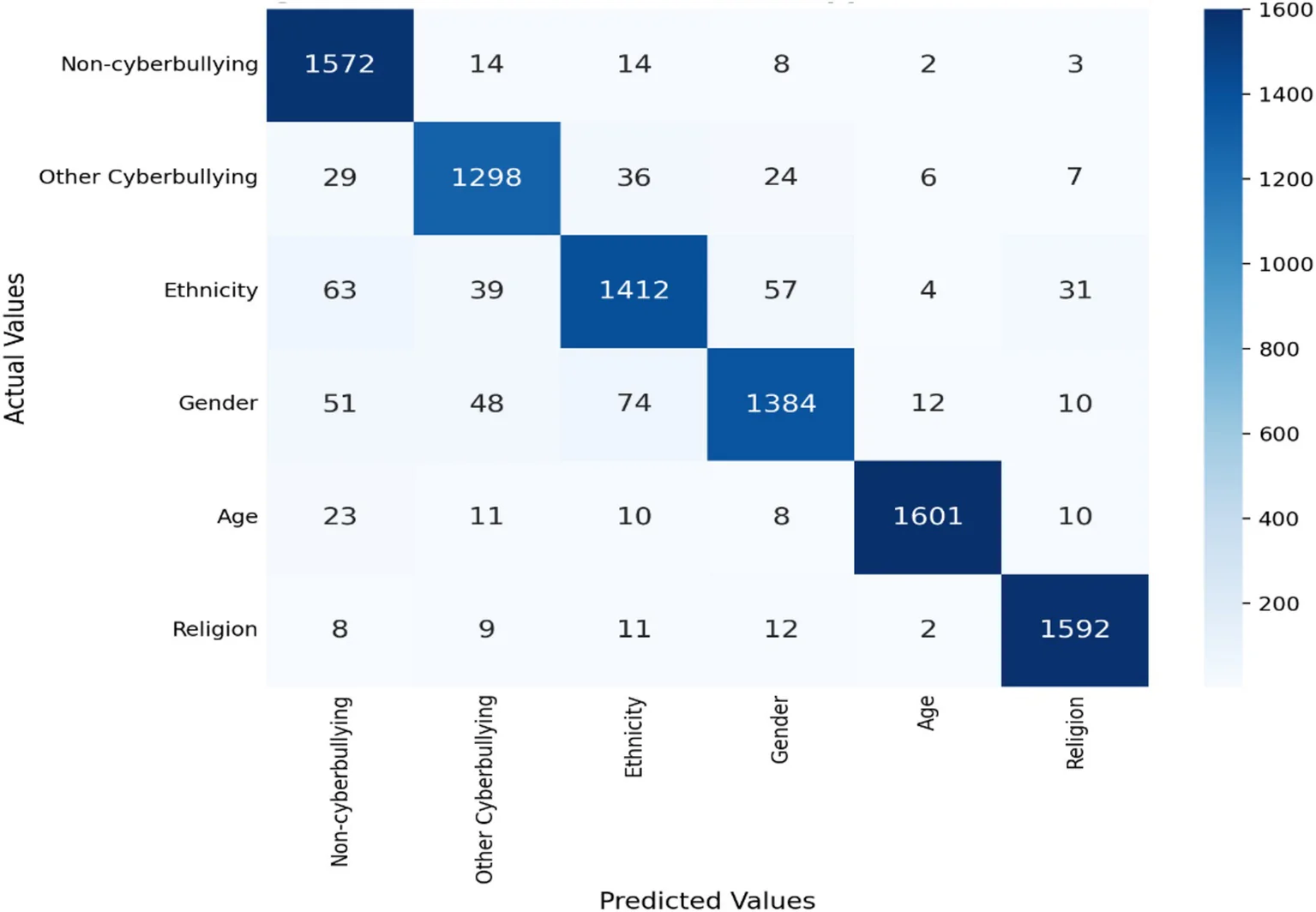
Six-class confusion matrix of the Support Vector Machine classifier across all cyberbullying categories.

**Figure 10:**
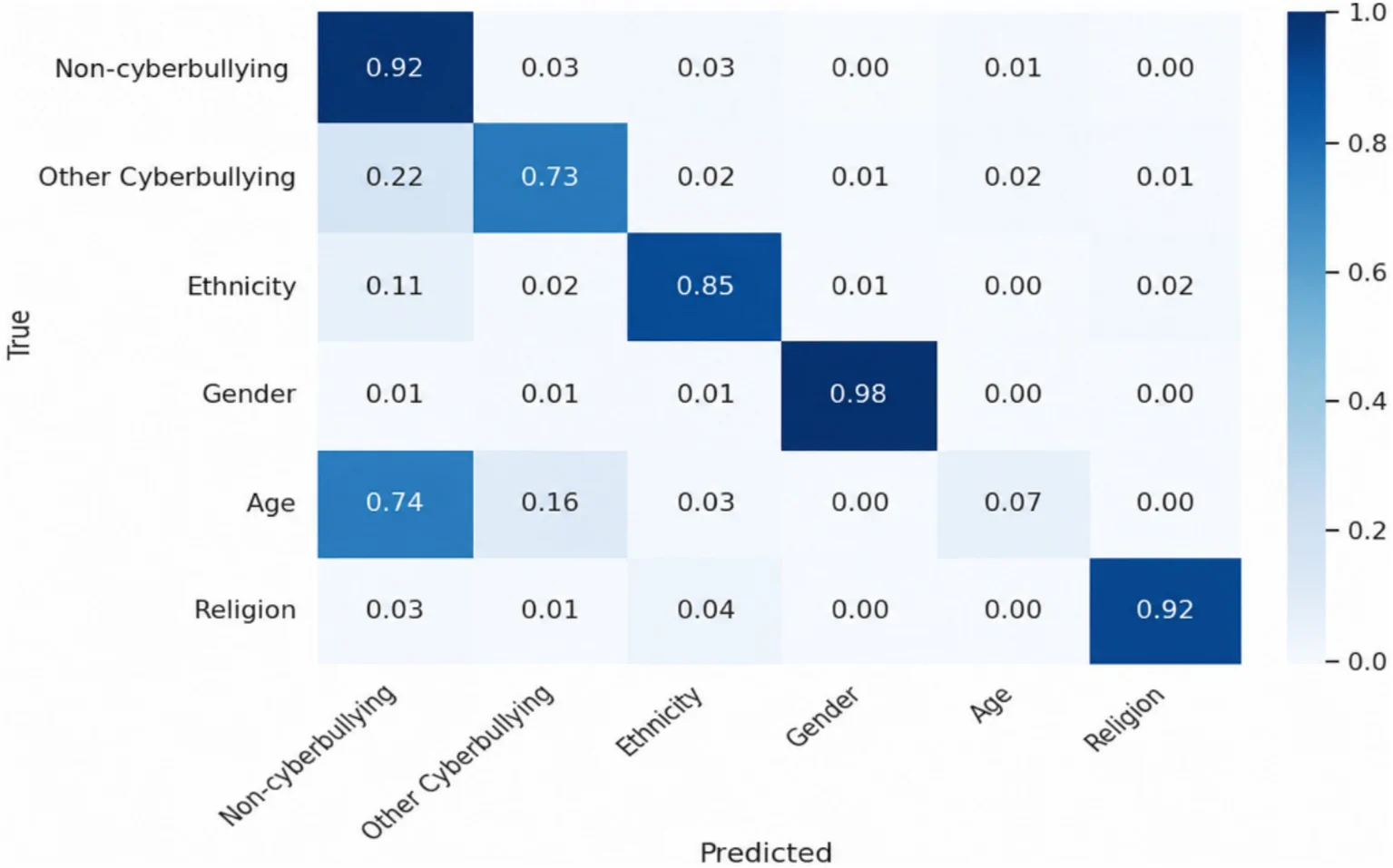
Confusion matrix of the BiLSTM model.

**Figure 11:**
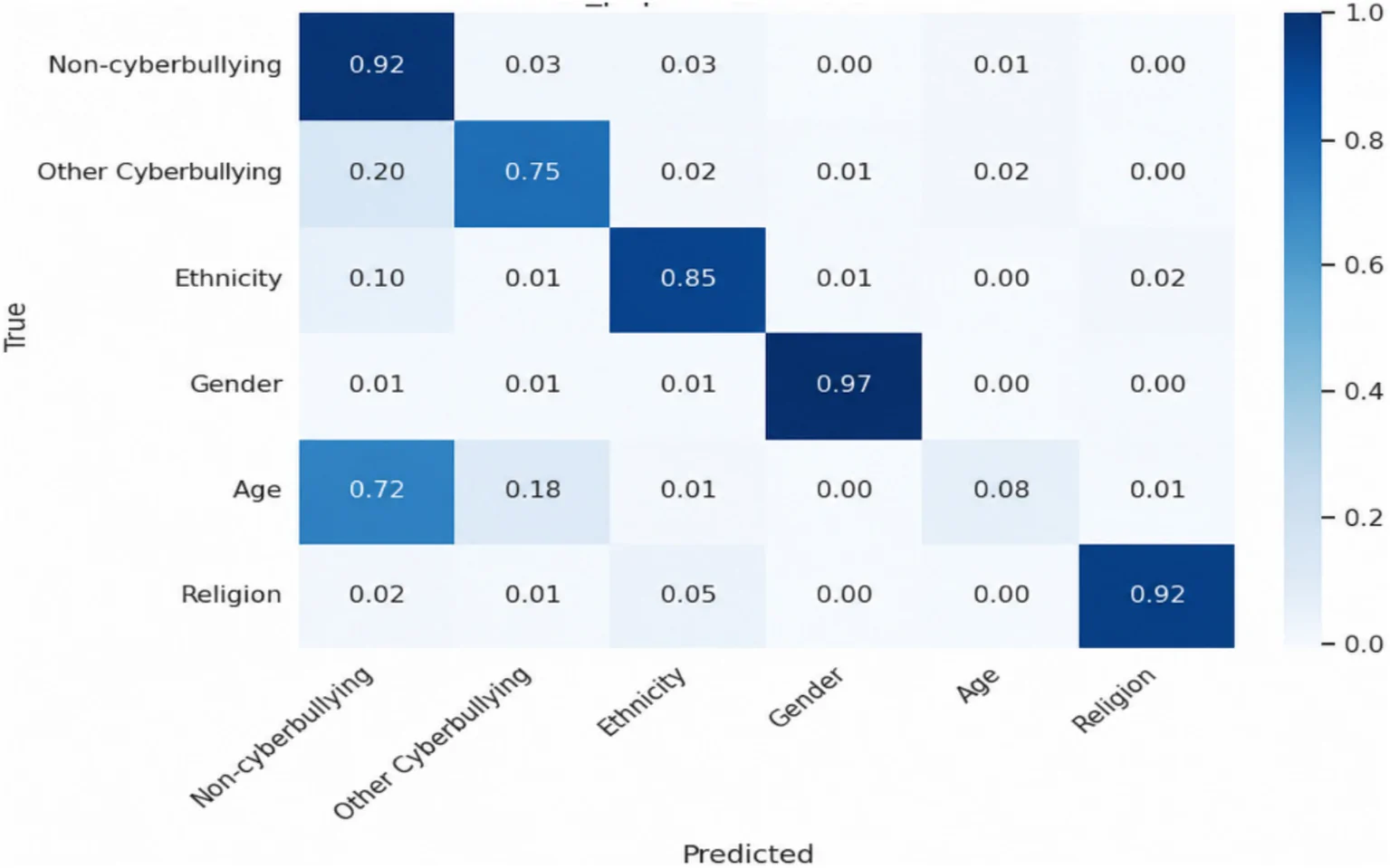
Confusion matrix of the BiGRU model.

The confusion matrices show that all four models achieved high classification performance, although their error distributions differed across demographic categories. Among the traditional machine learning models, the Extra Trees Classifier produced the fewest misclassifications, demonstrating its ability to exploit discriminative TF-IDF features for separating cyberbullying classes. SVM also achieved strong performance but exhibited slightly greater confusion between categories with overlapping lexical patterns. A consistent source of error across all models was the gender and age categories. These classes frequently share similar vocabulary and contextual expressions, leading to overlapping decision boundaries. Since TF-IDF represents documents using independent term frequencies rather than semantic context, traditional machine learning models occasionally misclassified tweets containing gender-specific terms directed at younger individuals.

The BiLSTM and BiGRU models exhibited different classification characteristics. By learning contextual dependencies from sequential text, both models better captured implicit abusive language and semantically related expressions than TF-IDF-based classifiers. Nevertheless, confusion between the gender and age categories remained, indicating that these demographic classes often require deeper pragmatic understanding beyond lexical semantics. Another common challenge was the age category, which recorded the highest misclassification rate among the deep learning models. Unlike religion or ethnicity related cyberbullying, age related content is frequently expressed through indirect stereotypes, colloquial phrasing, or implicit references rather than explicit abusive terms. Such implicit expressions remain difficult for both statistical and neural models to classify accurately. From a culturally aware perspective, religion and ethnicity were consistently classified more accurately than gender and age. Identity-based cyberbullying targeting religion and ethnicity often contains explicit discriminatory vocabulary, enabling clearer decision boundaries. In contrast, gender- and age-related abuse commonly relies on subtle language, stereotypes, and contextual references, increasing classification ambiguity. To further quantify these observations, Table 8 summarizes the class-wise performance of the best-performing traditional machine learning and deep learning models.

**Table 8:** Class-wise performance comparison of the best machine learning and deep learning models.

Model	Class	Precision (%)	Recall (%)	F1-score (%)	Overall accuracy (%)
Extra Trees	Religion	99.37	95.50	97.39	97.79
	Age	98.61	98.02	98.31	
	Gender	94.51	97.74	96.10	
	Ethnicity	100.00	100.00	100.00	
	Other cyberbullying	96.57	97.26	96.91	
	Non-cyberbullying	97.71	98.32	98.01	
Support Vector Machine	Religion	96.31	97.43	96.87	93.30
	Age	98.40	96.27	97.33	
	Gender	92.70	87.65	90.10	
	Ethnicity	90.69	87.92	89.28	
	Other cyberbullying	91.47	92.71	92.09	
	Non-cyberbullying	90.03	97.46	93.60	
BiLSTM	Religion	85.26	91.81	88.42	90.50
	Age	62.77	67.01	63.97	
	Gender	92.33	97.88	95.02	
	Ethnicity	68.42	84.65	75.67	
	Other cyberbullying	58.90	72.91	65.16	
	Non-cyberbullying	96.95	92.30	94.57	
BiGRU	Religion	83.24	91.94	87.37	90.49
	Age	62.80	67.64	64.10	
	Gender	91.81	96.95	94.31	
	Ethnicity	68.12	85.42	75.80	
	Other cyberbullying	59.66	74.94	66.43	
	Non-cyberbullying	97.18	92.11	94.58	

Table 8 confirms the trends observed in the confusion matrices. Extra Trees achieved the highest precision, recall, and F1-score for categories characterized by explicit lexical cues, particularly religion, ethnicity, and age. SVM produced comparable performance but showed slightly lower recall across several demographic categories, reflecting greater sensitivity to overlapping lexical features. Among the deep learning models, BiLSTM and BiGRU achieved strong performance for gender, religion, and non-cyberbullying, demonstrating their ability to learn contextual representations from sequential text. However, both models struggled substantially with the age category, where precision and recall dropped well below the other five classes, indicating that implicit or context-dependent expressions in this category limited classification performance for both recurrent architectures. To further examine class level prediction behavior, six class confusion matrices were generated using the identical label space adopted during training and testing. The matrices include Religion, Age, Gender, Ethnicity, Other Cyberbullying, and Non-cyberbullying categories, allowing detailed analysis of inter-class confusion patterns. Extra Trees remains highly effective for detecting explicit demographic-related cyberbullying through discriminative lexical features, whereas BiLSTM and BiGRU provide stronger contextual understanding of implicit abusive language. These findings emphasize the importance of class-wise evaluation and suggest that integrating statistical feature representations with contextual neural architectures may further improve culturally aware cyberbullying detection.

### 4.5 Cultural, demographic sensitivity, and feature importance analysis

Table 9 summarizes the cultural characteristics of the best-performing traditional machine learning and deep learning models. The Extra Trees Classifier achieved the highest overall performance by effectively utilizing TF-IDF features to discriminate explicit lexical patterns, whereas the Support Vector Machine (SVM) also demonstrated strong performance in the high-dimensional feature space. In contrast, the BiLSTM and BiGRU models leveraged contextual sequence learning through word embeddings, enabling improved recognition of implicit and context-dependent abusive language. These findings indicate that traditional machine learning and deep learning models provide complementary strengths for culturally aware cyberbullying detection.

**Table 9:** Cultural and demographic behavior of the best-performing models.

Model	Feature representation	Strength	Cultural observation
Extra Trees	TF-IDF	Highest overall performance	Excellent detection of explicit religion- and ethnicity-related cyberbullying.
SVM	TF-IDF	Strong decision boundaries	Sensitive to overlapping demographic vocabulary.
BiLSTM	Word Embedding + BiLSTM	Contextual sequence learning	Better recognition of implicit and context-dependent abuse.
BiGRU	Word Embedding + BiGRU	Efficient contextual modeling	Similar contextual performance with lower computational cost.

As shown in Table 9, the traditional machine learning models achieved superior performance when cyberbullying contained explicit lexical indicators, whereas the deep learning models demonstrated greater capability in learning contextual information and semantic relationships. This complementary behavior suggests that statistical feature representations and contextual sequence learning capture different characteristics of abusive language and therefore contribute differently to culturally aware cyberbullying detection. A further examination of the classification results indicates that the Religion and Ethnicity categories consistently achieved higher precision, recall, and F1-score than the Gender and Age categories. This performance difference can be attributed to the lexical characteristics of the dataset. Religion- and ethnicity-related cyberbullying frequently contains explicit identity-related terms and offensive keywords, allowing TF-IDF to generate highly discriminative feature representations that are effectively exploited by tree-based and margin-based classifiers. Conversely, the Gender and Age categories rely more heavily on indirect insults, stereotypes, colloquial expressions, and context-dependent language. These linguistic patterns are distributed across multiple words rather than a small set of highly discriminative keywords, making them inherently more difficult to distinguish using lexical frequency-based representations. Although the BiLSTM and BiGRU models improved contextual understanding through sequential word representations, they also exhibited reduced performance for categories characterized by implicit abusive language, indicating that contextual modeling alone cannot completely resolve culturally nuanced cyberbullying. The results demonstrate that cyberbullying detection performance varies considerably across demographic categories. Categories characterized by explicit lexical cues, particularly Religion and Ethnicity, were consistently classified more accurately than categories relying on implicit or context-dependent language, such as Gender and Age. These findings reinforce the importance of evaluating cyberbullying detection systems using class-wise and culturally aware analyses, as overall performance metrics alone may conceal systematic weaknesses in detecting abuse directed toward specific demographic groups.

### 4.6 Limitations and future considerations

Although the proposed framework achieved strong performance in culturally aware cyberbullying detection, several limitations should be acknowledged. First, the TF-IDF representation used by the traditional machine learning models captures lexical frequency rather than semantic context, limiting its ability to recognize sarcasm, irony, idiomatic expressions, euphemisms, and other implicit forms of abuse. This limitation contributes to the higher misclassification rates observed in the Gender and Age categories, where offensive language is often expressed indirectly rather than through explicit keywords. Second, although the dataset is balanced across demographic categories, annotation of offensive language remains inherently subjective. Cultural background, linguistic interpretation, and contextual understanding may influence annotation decisions, potentially affecting model generalization across different online communities. Furthermore, the dataset contains only English-language tweets and does not include multilingual or code-switched content, limiting the generalizability of the proposed framework to culturally diverse social media environments. Finally, although the proposed deep learning models improve contextual representation compared with TF-IDF-based classifiers, both traditional machine learning and deep learning models continue to struggle with subtle forms of cyberbullying involving implicit language, sarcasm, and rapidly evolving online slang. Consequently, maintaining high detection performance requires periodic dataset updates and model retraining. Although transformer-based models have demonstrated state-of-the-art performance in cyberbullying detection, the primary objective of this study was to establish a unified comparison between traditional machine learning and lightweight deep learning models under a culturally aware evaluation framework. Accordingly, transformer-based models were considered beyond the scope of the present work and remain an important direction for future research. Future research should investigate larger multilingual and culturally diverse datasets, fairness-aware learning strategies, and hybrid architectures that combine interpretable statistical features with contextual deep learning models. Such approaches may further improve robustness, reduce demographic bias, and enhance the reliability of culturally aware cyberbullying detection systems in real-world applications.

## 5 Conclusion

This study presented a comprehensive and culturally aware evaluation framework for cyberbullying detection by comparing traditional machine learning and deep learning models on the IEEE DataPort Fine-Grained Balanced Cyberbullying Dataset. Unlike conventional studies that primarily emphasize overall classification accuracy, this work investigated model performance across demographic-sensitive cyberbullying categories, including age, gender, religion, and ethnicity, providing a more detailed understanding of classification behavior in culturally diverse online environments. The results demonstrated that the Extra Trees Classifier achieved the highest overall performance, obtaining 97.79% accuracy, 97.82% precision, and 97.80% F1-score, outperforming the remaining traditional machine learning and deep learning models. Among the deep learning approaches, BiLSTM and BiGRU achieved competitive performance by learning contextual representations from sequential text, particularly for semantically complex categories. Comparative analysis further revealed that traditional machine learning models were highly effective for detecting cyberbullying characterized by explicit lexical patterns, whereas deep learning models provided improved contextual understanding of implicit abusive language. The confusion matrix and class-wise analyses further demonstrated that classification performance varied across demographic categories. Categories such as ethnicity achieved the highest predictive performance which may be associated with the presence of more explicit identity-related lexical patterns within the dataset. While gender remained the most consistently challenging category across all evaluated models due to its reliance on implicit, contextual, and overlapping linguistic expressions. Age showed divergent behavior across model types: tree-based models classified it accurately, while the recurrent deep learning architectures struggled substantially with this category, suggesting that age-related cyberbullying may depend more heavily on architecture-specific contextual modeling than on lexical cues alone. These findings highlight that overall accuracy alone is insufficient for evaluating culturally aware cyberbullying detection systems and emphasize the importance of class-wise and demographic-sensitive performance analysis. This study demonstrates that combining traditional machine learning and deep learning provides valuable insights into the strengths and limitations of different modeling paradigms for cyberbullying detection. While TF-IDF-based machine learning models remain computationally efficient and highly interpretable, deep learning models offer improved contextual representation for semantically complex online abuse. Future work should investigate hybrid architectures, multilingual and culturally diverse datasets, fairness-aware learning strategies, and continual model adaptation to improve robustness, reduce demographic bias, and enhance the practical deployment of culturally aware cyberbullying detection systems.

## Data Availability

The datasets presented in this study can be found in online repositories. The names of the repository/repositories and accession number(s) can be found at: https://ieee-dataport.org/open-access/fine-grained-balanced-cyberbullying-dataset.
